# ZINC-INDUCED FACILITATOR-LIKE family in plants: lineage-specific expansion in monocotyledons and conserved genomic and expression features among rice (*Oryza sativa*) paralogs

**DOI:** 10.1186/1471-2229-11-20

**Published:** 2011-01-25

**Authors:** Felipe K Ricachenevsky, Raul A Sperotto, Paloma K Menguer, Edilena R Sperb, Karina L Lopes, Janette P Fett

**Affiliations:** 1 Centro de Biotecnologia, Universidade Federal do Rio Grande do Sul, Av. Bento Gonçalves 9500, P.O.Box 15005, Porto Alegre, 91501-970, Brazil; 2 Departamento de Botânica, Instituto de Biociências, Universidade Federal do Rio Grande do Sul, Av. Bento Gonçalves 9500, Porto Alegre, 91501-970, Brazil

## Abstract

**Background:**

Duplications are very common in the evolution of plant genomes, explaining the high number of members in plant gene families. New genes born after duplication can undergo pseudogenization, neofunctionalization or subfunctionalization. Rice is a model for functional genomics research, an important crop for human nutrition and a target for biofortification. Increased zinc and iron content in the rice grain could be achieved by manipulation of metal transporters. Here, we describe the *ZINC-INDUCED FACILITATOR-LIKE (ZIFL) *gene family in plants, and characterize the genomic structure and expression of rice paralogs, which are highly affected by segmental duplication.

**Results:**

Sequences of sixty-eight *ZIFL *genes, from nine plant species, were comparatively analyzed. Although related to MSF_1 proteins, ZIFL protein sequences consistently grouped separately. Specific ZIFL sequence signatures were identified. Monocots harbor a larger number of *ZIFL *genes in their genomes than dicots, probably a result of a lineage-specific expansion. The rice *ZIFL *paralogs were named *OsZIFL1 *to *OsZIFL13 *and characterized. The genomic organization of the rice *ZIFL *genes seems to be highly influenced by segmental and tandem duplications and concerted evolution, as rice genome contains five highly similar *ZIFL *gene pairs. Most rice *ZIFL *promoters are enriched for the core sequence of the Fe-deficiency-related box IDE1. Gene expression analyses of different plant organs, growth stages and treatments, both from our qPCR data and from microarray databases, revealed that the duplicated *ZIFL *gene pairs are mostly co-expressed. Transcripts of *OsZIFL4*, *OsZIFL5*, *OsZIFL7*, and *OsZIFL12 *accumulate in response to Zn-excess and Fe-deficiency in roots, two stresses with partially overlapping responses.

**Conclusions:**

We suggest that *ZIFL *genes have different evolutionary histories in monocot and dicot lineages. In rice, concerted evolution affected *ZIFL *duplicated genes, possibly maintaining similar expression patterns between pairs. The enrichment for IDE1 boxes in rice *ZIFL *gene promoters suggests a role in Zn-excess and Fe-deficiency up-regulation of *ZIFL *transcripts. Moreover, this is the first description of the *ZIFL *gene family in plants and the basis for functional studies on this family, which may play important roles in Zn and Fe homeostasis in plants.

## Background

Duplications are recurrent in the evolutionary history of plant genomes. Whole genome duplications (or polyploidy) are described for dicotyledons and monocotyledons [1-4]. It is estimated that the incidence of polyploidy in angiosperms is 30-80%, and ploidy changes may represent about 24% of speciation events [5]. Duplication generates two copies of each gene, and the fate of duplicated genes was first described by Ohno: one copy should maintain the ancient function and another copy should lose function (pseudogenization) or gain a new function (neofunctionalization) [6]. This model was improved, giving rise to the duplication-degeneration-complementation (DDC) model, where the duplicated copies can have complementary functions that resemble the ancestral gene's function (subfunctionalization) [7]. The DDC model's predictions are believed to be more accurate than the previous model, since loss-of-function changes in regulatory regions are more likely to occur than gain-of-function mutations [7]. Other improvements of the basic model for duplicated gene retention, involving buffering of crucial functions via conversion and crossing-over, were recently proposed [8,9].

Due to repetitive genome duplications, plants are likely to harbor relatively larger gene families, as compared to animal genomes [10]. It is well established that one whole-genome duplication occurred in the cereal lineage, estimated 70 million years ago (MYA), preceding the radiation of the major cereal clades by 20 million years or more [3,11]. Recently, comparing the genomic sequences of rice (*Oryza sativa*) and *Sorghum bicolor*, it was demonstrated that an early duplication occurred in the monocot lineage [4]. The duplication blocks cover at least 20% of the cereals transcriptome [4]. It was also shown that expression divergence between duplicate genes is significantly correlated with their sequence divergence [12]. After duplication, gene pairs rapidly diverge, and only a small fraction of ancient gene pairs do not show expression divergence [12]. However, for some genomic segments, concerted evolution homogenizes homologous sequences through unequal crossing-over and gene conversion, changing the estimated duplication age and gene divergence [9,13-15].

Rice was first described as having 18 duplicated segments which cover 65.7% of its genomic sequence, and several individual gene duplications [16]. More recent estimates account for 29 duplications in the rice genome, including 19 minor blocks that overlap with 10 major blocks [17]. A duplication block between chromosomes 11 and 12 has been extensively characterized in rice and other cereals, although the age of its birth is still controversial [9,14,15,18,19]. Rice is a model for cereal genomic and genetics studies, due to the availability of the genome sequences from two varieties, extensive gene annotation, and mutant resources [20-24]. Rice is also a major staple food, feeding nearly half of the world's population. However, it is a poor source of minerals such as iron (Fe) and zinc (Zn), the two mineral elements most commonly lacking in human diets [25,26]. Metal homeostasis in plants has been extensively studied in recent years, with a special focus on the transition metals Zn and Fe [27-29]. Thus, rice emerges both as a model species for physiological and molecular studies and as a candidate for biotechnological improvement aiming at Zn and Fe biofortification [30-32].

Both Zn and Fe are essential to mineral nutrition of plants. Zn has a key role in gene expression, cell development and replication, while Fe is necessary for photosynthesis, electron transport and other redox reactions [33]. Although essential, both can be toxic when in excess [34-37]. Several transporters involved in uptake and translocation inside the plant were described for Fe and Zn [35,38-43].

The *ZINC-INDUCED FACILITATOR 1 *gene (*AtZIF1*), described by Haydon and Cobbett, belongs to a new family of transporters, with three members in *Arabidopsis thaliana*: *AtZIF1 *(AT5G13740), *AtZIFL1 *(AT5G13750) and *AtZIFL2 *(AT3G43790) [34]. The AtZIF1 transporter is clearly involved in Zn homeostasis, as the loss-of-function *atzif1 *mutant has altered Zn distribution and its transcription is up-regulated by Zn-excess [34]. Importantly, AtZIF1 proteins are expressed in the tonoplast, and probably are involved in transport of Zn, Zn and a ligand or a ligand alone, to the vacuole [34]. Besides AtZIF1, only one similar protein had been previously characterized: the maize (*Zea mays*) Zm-mfs1, which is induced by infection by the pathogens *Cochliobolus heterostrophus *and *C. carbonum *and to ultraviolet light [44]. This gene is highly expressed in the *Les9 *disease lesion mimic background and in plant tissues engineered to express flavonoids or the avirulence gene avrRxv [44]. Both AtZIF1 and Zm-mfs1 are part of the Major Facilitator Superfamily (MFS), which comprises the largest superfamily of secondary transport carriers found in living organisms and is subdivided in at least 29 families [45]. More recently, *AtZIF1 *and *AtZIFL1 *were described as quantitative trait loci (QTL) candidates for Zn concentrations in *Arabidopsis *seeds [46]. In barley (*Hordeum vulgare*), microarray analyses revealed that a *ZIF1-like *gene is expressed in the aleurone layer of seeds and its transcription increases in the embryo upon foliar Zn application [47]. Therefore, it is possible that *ZIFL *genes are involved in Zn translocation to the seeds.

In this work, we describe the ZIF-like (ZIFL) family of transporters. We identified 68 family members from plants and reconstructed their phylogenetic relationships. We also analyzed in detail the organization of *ZIFL *genes in the rice (*Oryza sativa*) genome: the motif composition, genomic organization, and promoter sequences. We analyzed the expression of *OsZIFL *genes in different plant organs and developmental stages, as well as in response to different stresses. This is the first attempt to describe the *ZIFL *gene family in plants, and the first expression analysis of these genes in rice.

## Results

### ZIFL genes in plants

We first used the *AtZIF1*, *AtZIFL1 *and *AtZIFL2 *sequences to query genomic databases to determine the distribution of this gene family among plant species. Two dicots, *Vitis vinifera *and *Populus trichocarpa*, one bryophyte, *Physcomitrella patens*, one lycophyte, *Selaginella moellendorffii*, and four monocots, *Sorghum bicolor*, *Brachypodium distachyon*, *Oryza sativa *and *Zea mays *had their genomes screened for *ZIFL *genes. All sequences found through this search plus the three *Arabidopsis *sequences were used to generate a Hidden Markov Model (HMM) profile to iteratively search the same genomes (see Methods). The final dataset consists of 66 genes coding for proteins already annotated (Additional File 1) and two unannotated proteins from *Zea mays *(Additional File 2).

All organisms queried contain *ZIFL *sequences, with predicted protein sequences ranging from 289 to 557 amino acids and an average of 468.4 amino acids per protein. All gene sequences begin with an initiation codon and end with a stop codon, except for the protein PpZIFL1, which lacks a small N-terminal portion (about 50 amino acids) and was included in the analyses. The overall structure contains 11 to 12 predicted transmembrane (TM) domains (Additional File 1 and Additional File 2), found in 63% of the proteins in our dataset. Fourteen putative proteins are predicted to have 10 TM domains, and 11 proteins have seven to nine TM domains (Additional File 1 and Additional File 2).

Dicot species have a small number of *ZIFL *gene copies, with *V. vinifera *and *P. trichocarpa *showing five and four paralogs of *ZIFL *in their genomes, similar to the three members of the *Arabidopsis ZIFL *gene family [34]. Conversely, monocot species show a higher number of *ZIFL *genes, with *S. bicolor *having the highest number of members (14), followed by rice (13), *B. distachyon *(10) and *Z. mays *(10). *P. patens *and *S. moellendorffii *harbor two and seven *ZIFL *genes, respectively. Clearly, monocot species have a higher number of *ZIFL *family paralogs than dicots. The seven *ZIFL *genes found in *S. moellendorffii *seem to be closely related and not originated from the same expansion which originated the monocot *ZIFL *genes.

### ZIFL proteins are a distinct family of MFS transporters

The ZIFL proteins are all part of the Major Facilitator Superfamily (MFS) clan of transporter proteins (Pfam number CL0015), composed by 22 families. They show similarity to the MFS_1 family (Pfam number PF07690), which is the largest family within the MFS clan. We used the MFS_1 HMM profile to isolate the MFS_1 proteins from all dicot and monocot genomes analyzed in this work. Phylogenetic trees reconstructing the evolutionary history of MFS_1 and ZIFL proteins for each species were generated using the neighbor-joining method (Additional File 3). We observed that in all cases the ZIFL proteins clustered in a separate group from all other MFS_1 members. This result could be an indication that ZIFL is a distinct family of MFS transporters.

**Figure 1:**
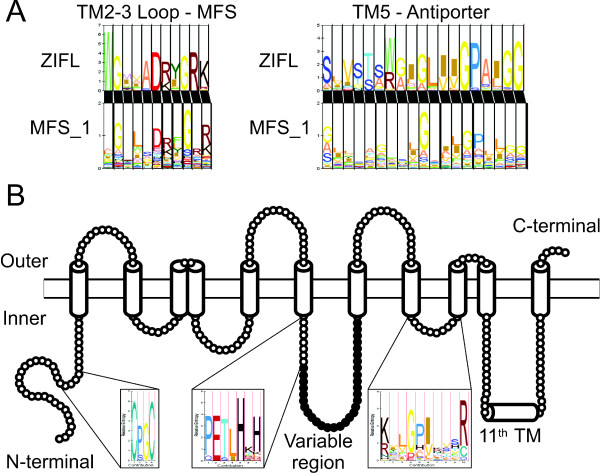
**ZIFL family sequence signatures**. (A) Alignment of ZIFL and MFS_1 signatures present in the cytoplasmic loop between TM2 and TM3 (MFS signature) and in TM5 (antiporter signature). (B) ZIFL specific signature not found in general MFS_1 proteins. The Cys motif C-[PS]-G-C is observed in the N-terminal cytoplasmic loop; the His motif [PQ]-E-[TS]-[LI]-H-x-[HKLRD] is in the cytoplasmic loop between TM6 and TM7, before the beginning of the variable region (in black); the [RK]-x(2)-G-P-[IV]-x(3)-R motif is in the cytoplasmic loop between TM8 and TM9. The overall transmembrane topology of the ZIFL proteins is schematically shown.

Simmons et al suggested that sequences similar to Zm-mfs1 (ZmZIFL5 in Additional File 1 and throughout this work) could be a distinct group of MFS proteins found in plants [44]. This was based on comparison of signature sequences of nine plant sequences to bacterial and fungal MFS sequences. To confirm this hypothesis, we searched for signatures in the ZIFL HMM profile and aligned them to the MFS_1 HMM profile. We found the canonical MFS signature, located in the cytoplasmic loop between TM2 and TM3, as well as the antiporter signature in TM5 (Figure 1A). When aligning these signatures to the MFS_1 HMM profile, we noticed that the ZIFL MFS signature G-x(3)-D-[RK]-x-G-R-[RK] has a conserved tryptophan (W) before the first glycine position, which is not observed in MFS_1 (Figure 1A). The antiporter signature, S-x(8)-G-x(3)-G-P-x(2)-G-G, is also slightly different, having preference for serine in the first position, instead of glycine, as observed by Simmons et al (Figure 1A) [44]. The presence of these conserved positions indicates that ZIFL transporters share structural and functional similarities with MFS antiporters, although they show specific features that are not common to other MFS proteins.

**Figure 2:**
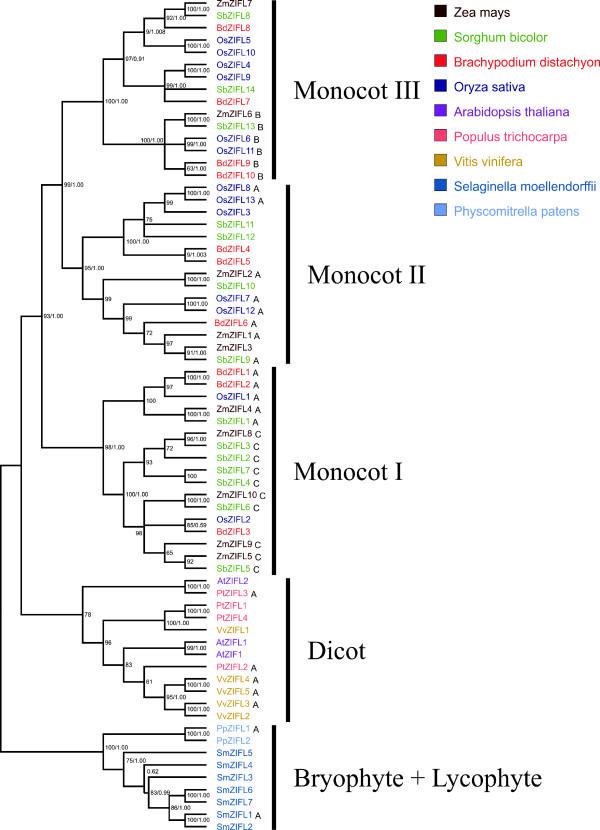
**Phylogenetic tree showing the relationships between ZIFL protein sequences**. The phylogenetic tree is based on a sequence alignment of 68 ZIFL members. The tree was generated with MEGA 4.1 software. Bootstrap values from 1,000 replicates using the neighbor-joining method and posterior probabilities from Bayesian analyses are indicated at each node when both methods agree with tree topology. Proteins showing motifs A, B or C within the variable region are indicated by capital letters.

The ZIFL sequences also show signatures that are not shared with MFS_1 proteins. The conserved positions in the loop between TM8 and TM9, [RK]-x(2)-G-P-[IV]-x(3)-R, previously reported by Simmons et al, were confirmed in our dataset with a few changes (Figure 2B) [44]. Importantly, we found two new conserved signatures that are specific for the ZIFL proteins. One of them is a cysteine (Cys)-containing motif C-[PS]-G-C in the cytoplasmic N-terminal loop of ZIFL proteins, and the second one is a histidine (His)-containing motif [PQ]-E-[TS]-[LI]-H-x-[HKLRD] in the cytoplasmic loop between TM6 and TM7, before the beginning of a variable region (Figure 2B; see below). From our dataset of 68 ZIFL proteins, 58 have the Cys motif, with only three proteins showing a serine residue in the second position instead of a proline (Additional File 4). For the histidine motif, 61 ZIFL proteins have the conserved residues (Additional File 4). From these, 45 have the most conserved residues P-E-T-L-H-x-H, while the other 16 ZIFL members contain the same motif with no more than one residue substitution (Additional File 4). Considering that the MFS_1 family has 56,680 proteins with very low overall similarity between them, and that ZIFL proteins share both high similarity and unique signatures, we suggest that ZIFL proteins comprise a distinct family of transporters.

### *ZIFL *gene expansion is lineage specific

To address the hypothesis of a lineage specific expansion of *ZIFL *genes in monocot species, we generated an alignment using the amino acid sequences of the 68 *ZIFL *genes found and reconstructed the phylogenetic relationships of these protein sequences using two methods: neighbor-joining and bayesian analysis (Figure 2). Although some nodes are not in agreement comparing the two methods, our bootstrap values and posterior probabilities support all the major nodes of the tree, indicating that the reported group relationships are reliable (Figure 2).

Proteins from bryophyte and lycophyte species grouped together, with paralogs from each species in a separate cluster. The ZIFL proteins from dicots also formed a distinct group (Figure 2). However, there was no clear separation into sub-groups of orthologous sequences within the dicots group (Figure 2). Species-specific gene duplications are observed in *Arabidopsis *(AtZIF1 and AtZIFL1), *V. vinifera *(VvZIFL2 and VvZIFL3; VvZIFL4 and VvZIFL5) and *P. trichocarpa *(PtZIFL1 and PtZIFL4) (Figure 2).

The ZIFL paralogs from monocot species were grouped in three distinct groups, named Monocot I, Monocot II and Monocot III. All three ZIFL protein groups from the monocots contain paralogs from the four species included in our analysis. The Monocot I group contains 17 ZIFL proteins, while the Monocot II and Monocot III groups contain 15 proteins each (Figure 2). Both the number of sequences found in monocot species and the tree topology strongly suggest that the *ZIFL *gene family experienced an expansion in the monocot lineage, and that the last common ancestor of the monocots already had *ZIFL *paralogs of the three groups (Figure 2). Thus, the split of the four monocot species used in this work probably occurred after the expansion of the *ZIFL *family observed in monocots, and this expansion is not shared with other plant lineages.

### *ZIFL *paralogs are unequally distributed in the rice genome

**Figure 3:**
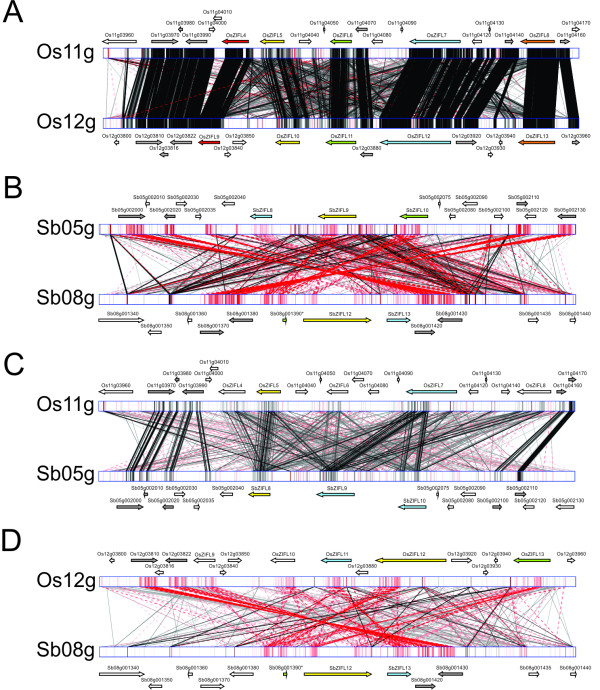
**Genomic alignment obtained with the GATA tool using 100 kb regions containing rice or *S. bicolor ZIFL *genes**. Black lines indicate a direct match, while red lines indicate an inverted match. Gene positions and orientations are denoted by arrows. *ZIFL *genes marked with the same color in both species indicate the closest homologs. Duplicated non-*ZIFL *genes are shown in gray. (A) Rice chromosomes 11 and 12. (B) *S. bicolor *chromosomes 5 and 8. (C) Rice chromosome 11 and *S. bicolor *chromosome 5. (D) Rice chromosome 12 and *S. bicolor *chromosome 8.

The identification of the *ZIFL *gene chromosome locations revealed that they are not evenly distributed in the rice genome, but rather arranged in clusters (Additional File 5). The same trend is observed in *S. bicolor *and *B. distachyon*, but not in *Z. mays *(Additional File 5). Rice *ZIFL *genes were named *ZIFL1 to 13 *based on their genomic locations. Two *ZIFL *genes, *OsZIFL1 *and *OsZIFL2 *are located in chromosome 1, and *OsZIFL3 *is located in chromosome 7. *OsZIFL4*, *OsZIFL5*, *OsZIFL6*, *OsZIFL7 *and *OsZIFL8 *are found in chromosome 11, while *OsZIFL9*, *OsZIFL10*, *OsZIFL11*, *OsZIFL12 *and *OsZIFL13 *are located in chromosome 12. Interestingly, the *ZIFL *genes arranged in tandem in chromosomes 11 and 12 are closely related, with *OsZIFL4 *being very similar to *OsZIFL9 *(92% of identity), *OsZIFL5 *to *OsZIFL10 *(95%), *OsZIFL6 *to *OsZIFL11 *(82%), *OsZIFL7 *to *OsZIFL12 *(85%) and *OsZIFL8 *to *OsZIFL13 *(73%) (Table 1). We used the GATA tool to align the 100 kb regions that include *OsZIFL *genes in chromosomes 11 and 12 (hereafter Os11 and Os12; Figure 3A). The regions of chromosomes 11 and 12 where these genes are located have already been described as a recent segmental duplication in the rice genome, what would explain the high number of matches between these regions (Figure 3A) [18,48]. However, the same duplication was recently found in *S. bicolor*, indicating that this segmental duplication is ancient to the split of these species [14,15]. We observed that *S. bicolor *chromosomes 5 and 8 (hereafter Sb05 and Sb08), which are homologous to rice chromosomes 11 and 12 (Os11 and Os12), harbor three and two *ZIFL *genes, respectively (Figure 3B) [14]. An incomplete sequence related to *ZIFL *is also found in chromosome 8 (Sb08g001390; Figure 3B). It is possible to observe that an inversion has occurred when comparing the orientation of *ZIFL *regions in Sb05 and Sb08 (Figure 3B). The alignment between rice and *S. bicolor *homologous chromosomes Os11 with Sb05 and Os12 with Sb08 demonstrate that the *S. bicolor ZIFL *region in Sb08 is inverted, since the alignment of Os11 with Sb05 is in direct orientation (Figure 3C) while the alignment of Os12 with Sb08 is in reverse (Figure 3D). Therefore, although in homologous regions, the *ZIFL *gene cluster in Sb08 is differentially oriented in relation to rice.

**Table 1:** Rice ZIFL sequence identity at the amino acid level.

	OsZIFL1	OsZIFL2	OsZIFL3	OsZIFL4	OsZIFL5	OsZIFL6	OsZIFL7	OsZIFL8	OsZIFL9	OsZIFL10	OsZIFL11	OsZIFL12
**OsZIFL2**	57											
**OsZIFL3**	44	44										
**OsZIFL4**	53	53	49									
**OsZIFL5**	55	55	50	74								
**OsZIFL6**	51	52	46	66	69							
**OsZIFL7**	51	52	43	56	58	50						
**OsZIFL8**	48	47	60	54	54	48	47					
**OsZIFL9**	54	55	44	**92**^**a**^	72	60	53	49				
**OsZIFL10**	55	52	48	72	**95**^**a**^	68	57	53	70			
**OsZIFL11**	47	49	42	61	62	**82**^**a**^	49	44	59	62		
**OsZIFL12**	54	54	56	62	66	58	**86**^**a**^	61	62	65	54	
**OsZIFL13**	40	37	40	39	40	35	48	**73**^**a**^	39	40	39	47

^a ^Rice ZIFL gene pairs from chromosomes 11 and 12

### *OsZIFL *genes organization is highly conserved

**Figure 4:**
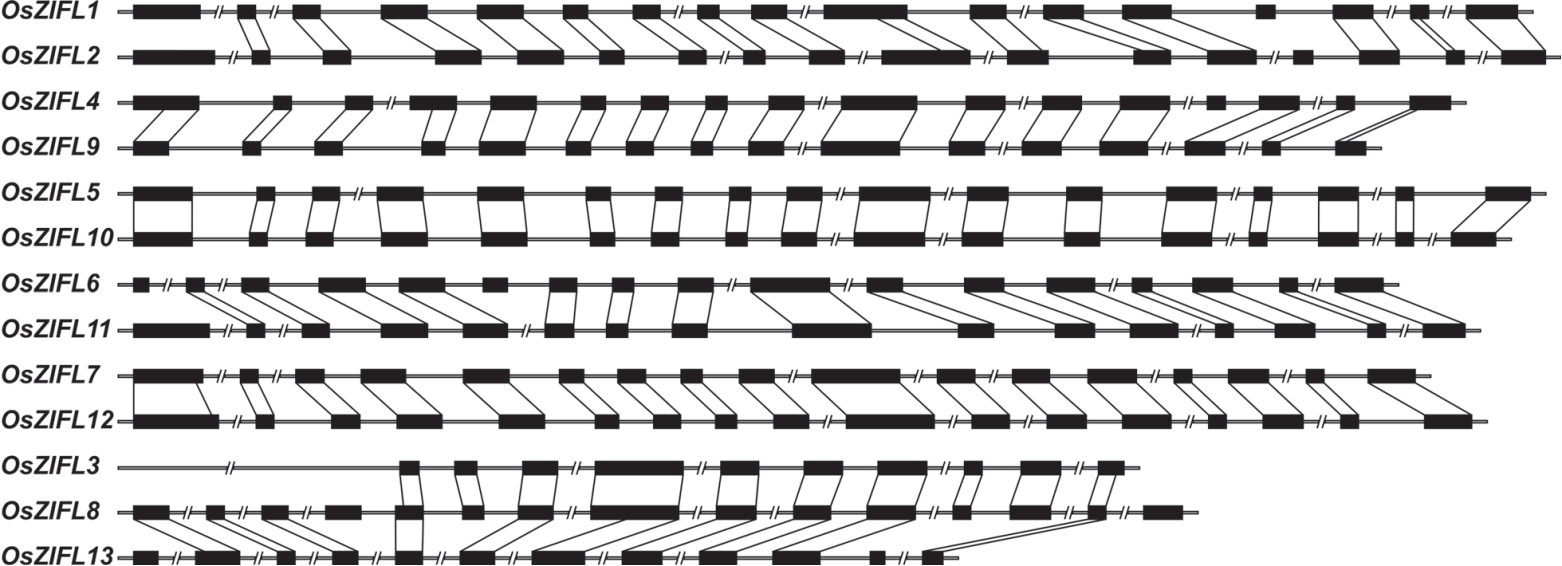
**Exon-intron gene organization of rice *OsZIFL *genes**. Exons are indicated with a black box and introns are indicated with lines. Introns with more than 200 bp are out of scale and indicated by an interrupted line. Exons from duplicated genes are linked with a black line.

We aligned the genomic and coding sequence (CDS) of each *ZIFL *gene from rice and determined the exon-intron organization (Figure 4). The exon sizes of each gene pair, *OsZIFL4*-*OsZIFL9*, *OsZIFL5*-*OsZIFL10*, *OsZIFL6*-*OsZIFL11*, *OsZIFL7*-*OsZIFL12 *and *OsZIFL8*-*OsZIFL13 *are nearly identical, with very few variations in sequences. We observed that *OsZIFL1 *and *OsZIFL2 *are probably originated from duplication, since they share a similar exon-intron organization. However, their amino acid sequences are only 57% identical (Table 1). This duplication probably occurred in the common ancestor of monocots, as orthologs from *S. bicolor*, *B. distachyon *and *Z. mays *were found for both *OsZIFL1 *and *OsZIFL2 *(Figure 2). *OsZIFL3 *is suggested to be originated from a partial duplication of the *OsZIFL8*-*OsZIFL13 *pair last common ancestor (Figures 2 and 4), and shares more identities to *OsZIFL8 *sequence (60%) than to *OsZIFL13 *(40%). Thus, it is clear that duplications were of major importance in the *ZIFL *family expansion in rice, especially the segmental duplication observed in chromosomes 11 and 12.

### Protein motif composition reveals a variable region in the ZIFL family

**Figure 5:**
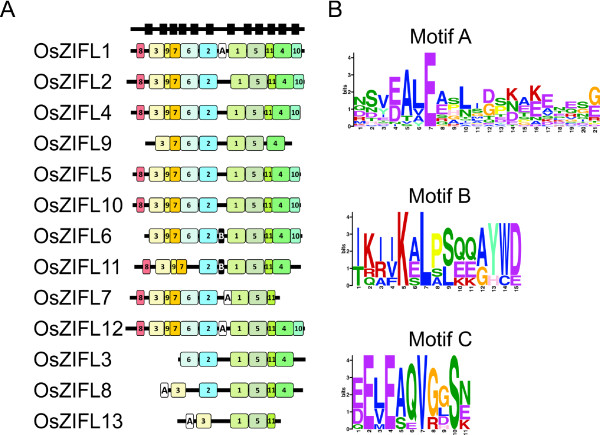
**Motifs in ZIFL protein sequences identified with the MEME tool**. (A) Conserved motifs in rice protein sequences encoded by *OsZIFL *genes. Motif numbers are according to table 2. Predicted transmembrane positions are shown as wide black boxes at the top. (B) Conserved motifs A, B and C present in the variable region of plant ZIFL proteins. Letters denote amino acids and wider letters indicate more conserved amino acids in the respective positions.

We aligned the 13 rice ZIFL proteins and observed that they share large similarity (Additional File 6 and Table 1). To search for functional sites shared by OsZIFL putative proteins, we used MEME (http://meme.nbcr.net/) to identify conserved motifs in their amino acid sequences [49]. We found eleven motifs shared by almost all 13 OsZIFL proteins, with few exceptions (Table 2, Figure 5A). Seven motifs matched the general MFS_1 motif in InterProScan (http://www.ebi.ac.uk/Tools/InterProScan/) (motifs 1, 2, 4, 5, 6, 7 and 9), while four showed no hits (motifs 3, 8, 10, and 11) (Table 2). The ZIFL signatures Cys motif and His motif are located within the motif 8 and motif 2, respectively (Table 2).

**Table 2:** Conserved motifs found in ZIFL protein sequences.

Motif	Width	Assign	Amino acid sequence
1	50	MFS^a^	NWPLMSSIILYCVFSFHDMAYSEIFSLWAESDRKYGGLSFSSEDVGQVLA
2	50	MFS^a^	QPAEKYPNVFSEKSIFGRFPYFLPCLCISVFAAVVLISCIWLPETLHKHK
3	41	No hit	LPISSLFPFLYFMIRDLHVAKREEDIGFYAGFVGASYMIGR
4	50	MFS^a^	LQNNAVPQDQRGTANGIATTAMSFFKAIAPAGAGVLFSWAQKRQHAAFFP
5	50	MFS^a^	GASLLVYQLFIYPWVHKVLGPINSSRIAAILSIPILCTYPFMTHLSGPWL
6	50	MFS^a^	RFLLGALNGMLGPIKAYSIEVCRPEHQALGLSIVSTAWGIGLVVGPAIGG
7	29	MFS^a^	PVIVFSIFSVVIFNTLFGLSTKYWMAITT
8	21	No hit	HDGCPGCAMERRKEEHKGIPY
9	15	MFS^a^	ASIFWGIVADRIGRK
10	28	No hit	GDQMVFFMLNVTEVIGLMLTFKPFLAVP
11	21	No hit	VLNIASMMKNNLAVTIITGTN
A^b^	21	No hit	NSVEALEEHLMDPNEEENENE
B^b^	15	No hit	IKRIKELPSQQAYWD
C^b^	11	No hit	EELEAQVGGSN

^a^MFS (Major Facilitator Superfamily Antiporter)

^b^Analysis performed with whole set of ZIF proteins (68 sequences)

OsZIFL1, OsZIFL2, OsZIFL4, OsZIFL5, OsZIFL10 and OsZIFL12 have all eleven motifs, while the duplicated pair OsZIFL8-OsZIFL13 and their duplicated copy in chromosome 7 (OsZIFL3) lack several motifs (Figure 5A). Some of these motifs are located in regions predicted to be transmembrane (Figure 5A, black boxes at the top). Further characterization is needed to determine if the duplicated rice ZIFL genes are becoming pseudogenes or acquiring new functions.

The OsZIFL4 duplicated copy OsZIFL9 lacks the N-terminal motif 8 and the C-terminal motif 10; OsZIFL6 lacks motif 8 and its duplicated copy OsZIFL11 lacks motif 6 and motif 10; the duplicated pair OsZIFL7 and OsZIFL12 only differ by the C-terminal motifs 4 and 10, which are absent in OsZIFL7 (Figure 5A). These differences suggest a divergence process between duplicated pairs. Moreover, it is clear that the central motifs are more conserved than those located at the N- and C-terminal regions of OsZIFL proteins (Figure 5A).

We also observed a variable region between motifs 1 and 2 which did not show significant pattern conservation in OsZIFL proteins (Figure 5A). This region is located between transmembrane regions 6 and 7 (considering 12 TM proteins) and is a cytoplasmic loop according to Conpred II predictions (Figure 1B). The variable region is preceded by the conserved His motif P-E-T-L-H-x-H (Figure 1B). Variable regions are found in transporters and could be involved in transport or sensing functions [50,51]. The whole set of 68 ZIFL proteins used in this work was submitted to MEME analysis to find any conserved motifs specifically in the variable region. Three motifs were found in this region and named motifs A, B and C (Table 2; Figure 5B). None matched any known motif in the InterPro database (Table 2). We indicated proteins that contain each motif in our phylogenetic tree (Figure 2) and showed their positions in rice ZIFL protein sequences (Figure 5A). Rice ZIFL proteins contain motifs A and B in their variable region, but not motifs C.

Motif A is present in proteins from the Monocot I, Monocot II, Dicot and Bryophyte-Lycophyte groups (Figure 1). This motif shows low amino acid conservation (Figure 5B). The negatively charged glutamic acid (E) residue in the seventh position of the motif is the most conserved residue. Conserved negatively charged residues are also found in the fourth position (glutamic or aspartic acid, E or D). Between these positions, two non-polar residues, alanine (A) and leucine (L) are also conserved (Figure 5B). Other positions containing a positively-charged residue of lysine (K), a negatively charged glutamic acid (E), and residues of leucine (L) and glycine (G), although less conserved, are present (Figure 5B). Charged positions could be involved in transporter specificity, as already described for cation diffusion facilitator (CDF) proteins [52]. Motif B is shared only by a sub-group of six proteins from monocot II (Figure 2). The fifth and seventh positions of this motif contain one positively charged residue and one hydrophobic residue, lysine (K) and leucine (L) (Figure 4B). Polar residues of serine (S), glutamine (Q) and tyrosine (Y), non-polar tryptophan (W) and proline (P) are also observed (Figure 4B). The motif C is common to 10 proteins from the Monocot I group (Figure 2), and is similar to motif A, showing the two glutamic acids (E) separated by one instead of two non-polar residues (Figure 5B). However, since only a small number of proteins share motifs B and C, we should be cautious on making assumptions about the functionality of conserved amino acids found in these motifs, as their conservation could be an effect of phylogenetic relatedness and not of evolutionary constraints.

Importantly, it is possible to observe the high divergence of the variable region even when comparing these three motifs. The variability is much higher in this region than in the whole sequence of ZIFL proteins, as MEME analysis revealed several motifs shared by all the 68 ZIFL proteins (data not shown). Therefore, these motifs in the cytoplasmic loop could be involved in specific functions of different ZIFL proteins.

### Expression of *OsZIFL *genes in rice vegetative and reproductive organs

**Figure 6:**
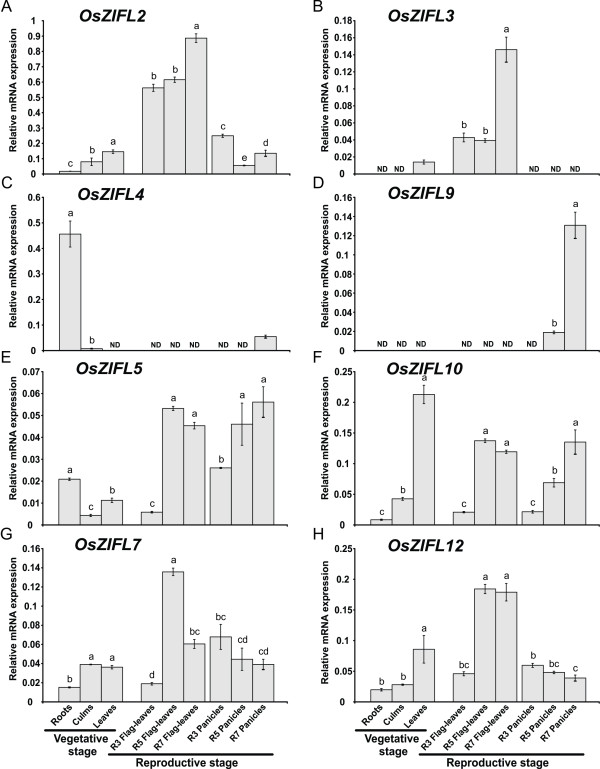
**Expression of *OsZIFL *genes in roots, culms and leaves during vegetative growth and flag leaves and panicles during reproductive growth, evaluated by qPCR**. Reproductive stages analyzed were R3 (panicle exertion), R5 (grain filling) and R7 (grain dry-down) from both flag leaves and panicles. (A) *OsZIFL2*. (B) *OsZIFL3*. (C) *OsZIFL4*. (D) *OsZIFL9*. (E) *OsZIFL5*. (F) *OsZIFL1*. (G) *OsZIFL7*. (H) *OsZIFL12*. Values are the averages of three samples ± SE. Different letters indicate that the mean values are different by the Tukey HSD test (P ≤ 0.05).

We analyzed the expression levels of *OsZIFL *transcripts in several rice organs by qPCR, including roots, culms and shoots (vegetative tissues); flag-leaves and whole panicles (reproductive tissues), both during R3 (panicle exertion), R5 (grain filling) and R7 (grain dry down) stages (Figure 6). Throughout our qPCR experiments, *OsZIFL1*, *OsZIFL6*, *OsZIFL8*, *OsZIFL11 *and *OsZIFL13 *transcripts were not detected or were detected below a confidence threshold for analysis. The expression levels of *OsZIFL *genes varied considerably, with some genes reaching higher expression levels (*OsZIFL2 *and *OsZIFL4*, Figures 6A and 6C) and others showing very low expression (*OsZIFL3*, *OsZIFL9*, *OsZIFL5 *and *OsZIFL7*; Figures 6B, 6D, 6E and 6G). *OsZIFL2 *and *OsZIFL3*, although not resultant of a duplication event, share a similar pattern of expression: both are more expressed in leaves and also accumulate in the later stages of flag-leaf development, reaching the highest levels in R7 (Figures 6A and 6B).

When analyzing gene pairs, we observed that *OsZIFL4 *is almost specifically expressed in roots, showing only little expression in panicles during the R7 stage (Figure 6C), while its duplicated copy *OsZIFL9 *is not expressed in vegetative tissues nor in flag-leaves, but is detected at low levels in panicles during R5 and at higher levels during R7 (Figure 6D). Transcripts from the *OsZIFL5*-*OsZIFL10 *pair show similar patterns of expression, especially when considering the reproductive organs flag-leaves and panicles (Figures 6E and 6F). *OsZIFL5 *and *OsZIFL10 *are both induced from R3 to R5 in flag-leaves, maintaining high levels at R7. In panicles, they are also induced from R3 to R5, although *OsZIFL10 *transcript levels are further induced from R5 to R7 (Figures 6E and 6F). In vegetative tissues, *OsZIFL5 *levels are higher in roots, while *OsZIFL10 *is more expressed in shoots (Figures 6E and 6F).

The genes from the *OsZIFL7*-*OsZIFL12 *pair also show similar expression patterns in the organs analyzed. *OsZIFL7 *is more expressed in culms and leaves, accumulates from R3 to R5 in flag-leaves and decreases its expression from R3 to R5 during panicle development (Figure 6G). The *OsZIFL12 *transcript accumulates in leaves and also increases from R3 to R5 in flag-leaves and decreases from R3 to R5 in panicles (Figure 6H). Taken together, our gene expression data demonstrates that, even after duplication and divergence, most *OsZIFL *genes still share similar expression patterns in rice organs within gene pairs.

### The Fe-deficiency element IDE1 is enriched in promoters of *OsZIFL *genes

**Figure 7:**
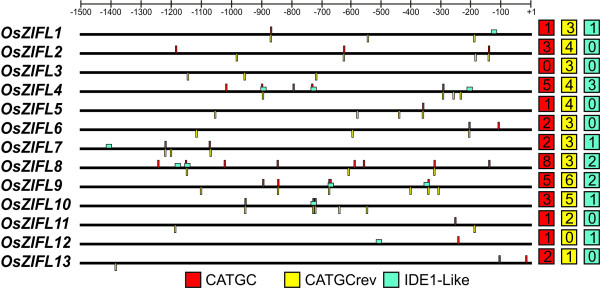
**Localization of CATGC and IDE1-like boxes in rice *OsZIFL *gene promoters**. Promoter sequences are shown from -1,500 to +1 position. CATGC boxes are shown as red (+ strand) or yellow (- strand) lines (CATGCrev); IDE1-like elements are shown as blue boxes. Total number of each box type from each promoter is shown at right.

To investigate the presence of conserved *cis*-elements in promoter regions of *OsZIFL *genes, we used the POCO tool [53]. This approach consisted in comparing the -1,500 to +1 regions of *OsZIFL *genes to several random samples of promoters from the entire *Arabidopsis *genome with the same size (each sample composed of 13 promoters). If a *cis*-element is more often found in the promoters of *OsZIFL *genes than in a random set of promoters, this *cis*-element is enriched in these sequences. The POCO analysis revealed that the sequence CATGC is enriched in our promoter set when compared to *Arabidopsis *promoters. This sequence is the core binding site of IDEF1 (iron-deficiency responsive element-binding factor 1), a transcription factor of the ABI3/VP1 family involved in Fe-deficiency response in rice [30,54]. As *Arabidopsis *is not closely related to rice and thus the motif frequency in promoters could vary between these species, we confirmed the enrichment by counting the average number of CATGC boxes in nearly 25,000 promoters of rice downloaded from Osiris (http://www.bioinformatics2.wsu.edu/cgi-bin/Osiris/cgi/home.pl) [55]. While the average number of the CATGC sequences in rice promoters was 3.24, in promoters of the thirteen *OsZIFL *genes it was 5.85 boxes per promoter. Some promoters are highly enriched for CATGC boxes, such as *OsZIFL2 *(7 boxes), *OsZIFL10 *(8 boxes), *OsZIFL4 *(9 boxes) and *OsZIFL9 *(10 boxes) (Figure 7). Genes that were not detected in our qPCR experiments such as *OsZIFL8 *and *OsZIFL1 *also have promoters enriched in CATGC boxes (11 and 6, respectively) (Figure 7). *OsZIFL5*, *OsZIFL6 *and *OsZIFL7 *promoters show 5 boxes each (Figure 7).

Since the CATGC box is the core motif of IDE1, we searched for IDE1-like sequences in promoters of *OsZIFL *genes following the method described by Kobayashi et al. [56]. We found eleven IDE1-like motifs distributed in seven gene promoters, *OsZIFL1*, *OsZIFL4*, *OsZIFL7*, *OsZIFL8*, *OsZIFL9*, *OsZIFL10 *and *OsZIFL12* (Figure 7). *OsZIFL4 *shows three sequences, two of them overlapping with CATGC boxes, while *OsZIFL8 *and *OsZIFL9 *show two IDE1-like motifs (Figure 7). Considering that the motif is 18 bp long, it is surprising to find such a high number of IDE1-like motifs in our promoter set. The enrichment for CATGC and IDE1-like sequences in promoters of *OsZIFL *genes suggests that they are possibly regulated by Fe-deficiency.

### Zn-excess and Fe-deficiency regulate *OsZIFL *expression mainly in rice roots

It has been demonstrated that *AtZIF1 *is up-regulated by Zn-excess in roots and leaves of *Arabidopsis *plants, as well as by Fe-deficiency [34,57,58]. As promoters of *OsZIFL *genes are enriched for Fe-deficiency *cis*-elements, we submitted rice plants to Zn-excess (200 μM) for three days and to Fe-deficiency (no Fe added to nutrient solution) for seven days. *OsZIFL *mRNA expression level was evaluated by qPCR in roots and leaves from both experiments.

**Figure 8:**
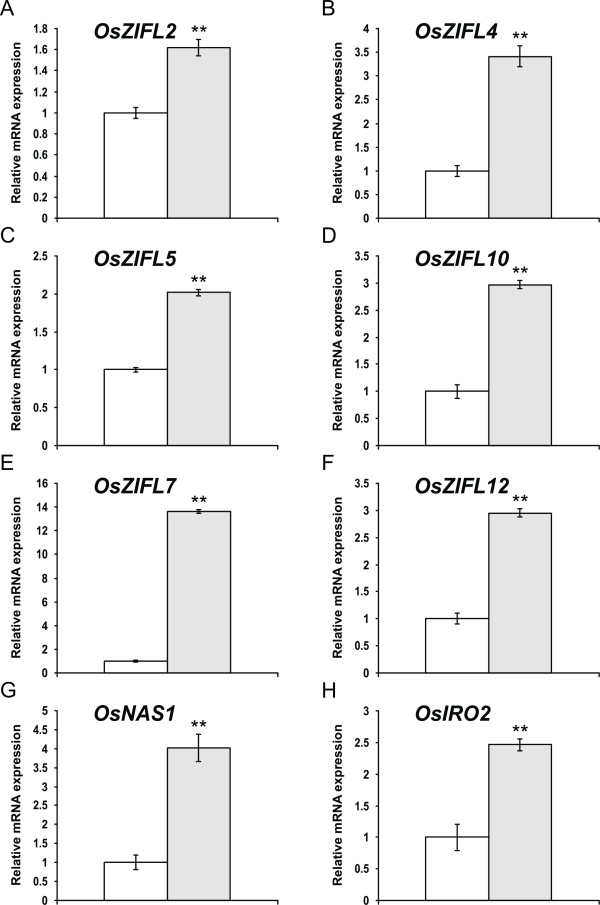
**Gene expression in roots of rice plants submitted for 3 days to 0.5 μM of Zn (control, white bars) or 200 μM of Zn (Zn+, gray bars), evaluated by qPCR**. (A) *OsZIFL2*. (B) *OsZIFL4*. (C) *OsZIFL5*. (D) *OsZIFL10*. (E) *OsZIFL7*. (F) *OsZIFL12*. (G) *OsNAS1*. (H) *OsIRO2*. Values are the averages of three samples ± SE. Statistical differences according to the Student's *t*-test in comparison to control are shown by one (p ≤ 0.05) or two asterisks (p ≤ 0.01).

Several *OsZIFL *genes were up-regulated in roots of Zn-excess treated plants: *OsZIFL2*, *OsZIFL4*, *OsZIFL5*, *OsZIFL10*, *OsZIFL7 *and *OsZIFL12 *(Figure 8). Expression of *OsZIFL1*, *OsZIFL3*, OsZIFL9 and of the duplicated pairs *OsZIFL6*-*OsZIFL11 *and *OsZIFL8*-*OsZIFL13 *was not detected. Expression of *OsZIFL4*, which is nearly root-specific (Figure 6C), is induced 3.5-fold by Zn-excess (Figure 8B). Both *OsZIFL5 *and *OsZIFL10*, a duplicated pair, are also up-regulated by 2- and 3-fold, respectively (Figures 8C and 8D). *OsZIFL7 *and *OsZIFL12 *show different patterns of induction, with *OsZIFL7 *induced by almost 14-fold in comparison to control levels (Figure 8E). *OsZIFL12*, although induced by Zn-excess in roots, is up-regulated only by 3-fold (Figure 8F). To confirm that our treatment was effective, we used *OsNAS1 *and *OsIRO2 *(Figures 8G and 8H), two genes up-regulated by Zn-excess in rice roots [59]. Therefore, the *OsZIFL *genes which are expressed in roots are up-regulated under Zn-excess.

**Figure 9:**
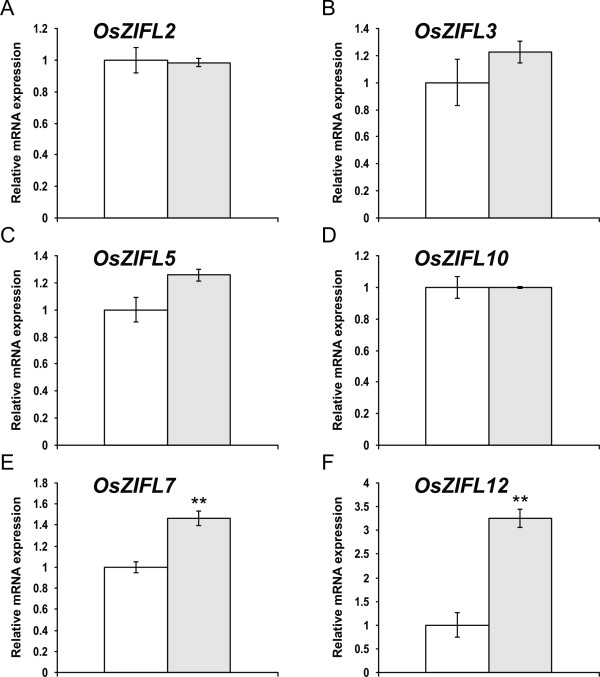
**Gene expression in leaves of rice plants submitted for 3 days to 0.5 μM of Zn (control, white bars) or 200 μM of Zn (Zn+, gray bars), evaluated by qPCR**. (A) *OsZIFL2*. (B) *OsZIFL3*. (C) *OsZIFL5*. (D) *OsZIFL10*. (E) *OsZIFL7*. (F) *OsZIFL12*. Values are the averages of three samples ± SE. Statistical differences according to the Student's *t*-test in comparison to control are shown by one (p ≤ 0.05) or two asterisks (p ≤ 0.01).

A very different expression pattern of *OsZIFL *genes was observed in leaves under Zn-excess: expression of *OsZIFL2*, *OsZIFL3*, *OsZIFL5 *and *OsZIFL10 *was not affected (Figures 9A, 9B, 9C and 9D). Only *OsZIFL7 *and *OsZIFL12 *mRNA levels were altered under Zn-excess: 1.4-fold and 3.3-fold higher than in the control treatment, respectively (Figures 9E and 9F). The *OsZIFL7 *and *OsZIFL12 *genes are a duplicated pair and are also up-regulated by Zn-excess in roots, suggesting a strong co-regulation under these conditions in both organs. However, most *OsZIFL *genes seem to be differentially regulated in leaves compared to roots when plants are under excessive Zn concentrations.

**Figure 10:**
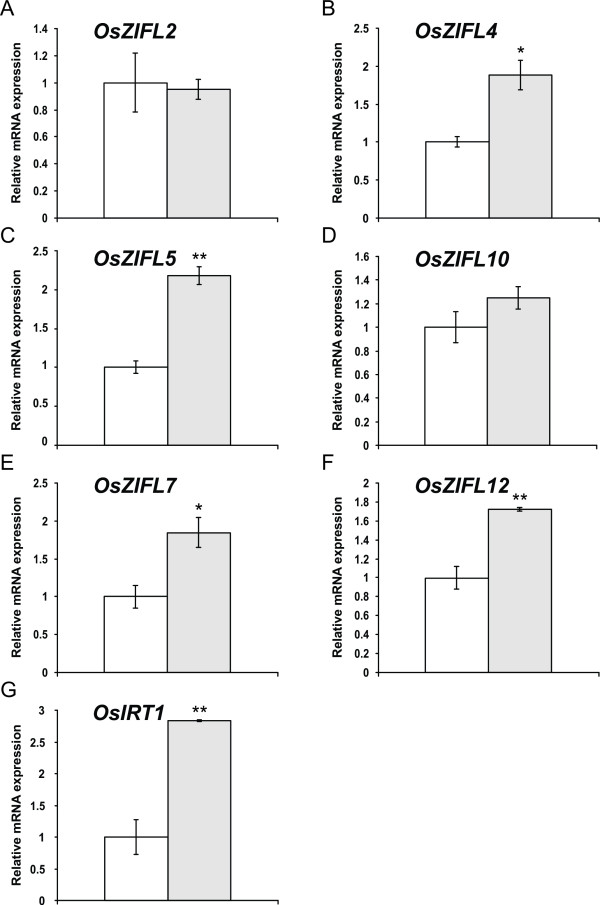
**Gene expression in roots of rice plants submitted for 7 days to control (100 μM Fe**^**+3**^**-EDTA, white bars) or Fe-deficiency (no Fe added, gray bars), evaluated by qPCR**. (A) *OsZIFL2*. (B) *OsZIFL4*. (C) *OsZIFL5*. (D) *OsZIFL10*. (E) *OsZIFL7*. (F) *OsZIFL12*. (G) *OsIRT1*. Values are the averages of three samples ± SE. Statistical differences according to the Student's *t*-test in comparison to control are shown by one (p ≤ 0.05) or two asterisks (p ≤ 0.01).

*OsZIFL *expression was also regulated in roots of plants under Fe-deficiency. Expression of *OsZIFL2 *and *OsZIFL10 *was not significantly increased by the treatment (Figures 10A and 10D). *OsZIFL4*, *OsZIFL5*, *OsZIFL7 *and *OsZIFL12*, however, were up-regulated by 1.8 to 2-fold (Figures 10B, 10C, 10E and 10F). This effect occurred in parallel with increased expression of *OsIRT1 *(2.8-fold), a gene already described as responsive to Fe-deficiency in rice roots [60,61]. This demonstrates that the plants were indeed under Fe-deficient conditions. Moreover, all four genes regulated by Fe-deficiency in roots were also induced by Zn-excess (Figure 8), confirming a trend for common responses to both stresses in this organ, as previously reported [59].

**Figure 11:**
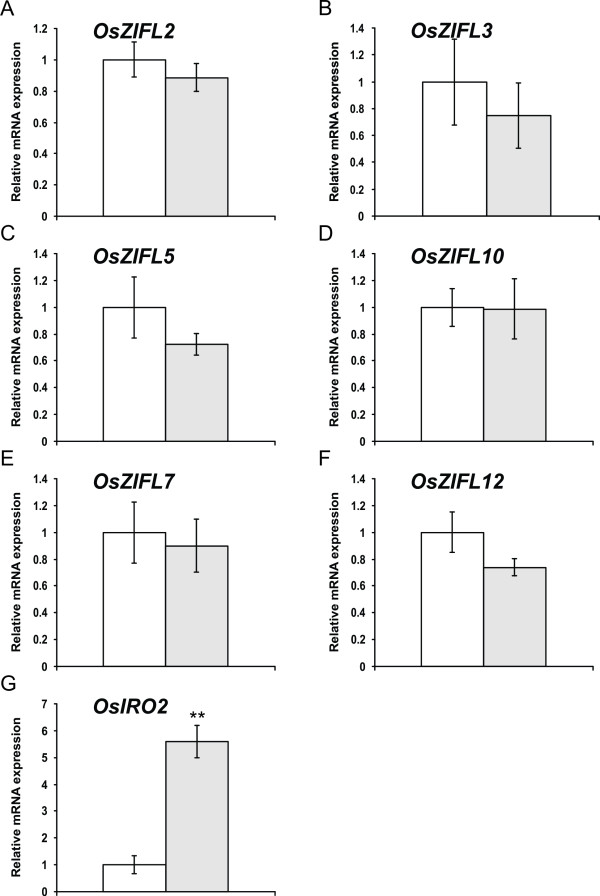
**Gene expression in leaves of rice plants submitted for 7 days to control (100 μM Fe**^**+3**^**-EDTA, white bars) or Fe-deficiency (no Fe added, gray bars), evaluated by qPCR**. (A) *OsZIFL2*. (B) *OsZIFL3*. (C) *OsZIFL5*. (D) *OsZIFL10*. (E) *OsZIFL7*. (F) *OsZIFL12*. (G) *OsIRO2*. Values are the averages of three samples ± SE. Statistical differences according to the Student's *t*-test in comparison to control are shown by one (p ≤ 0.05) or two asterisks (p ≤ 0.01).

A completely different response to Fe-deficiency was observed in leaves. None of the *OsZIFL *genes showed up-regulation under this condition (Figure 11A-F), although expression of the *OsIRO2 *gene, was up-regulated by 5.6-fold (Figure 11G). It is known that *OsIRO2 *is induced by Fe-deficiency in leaves [62]. This is, however, similar to *OsZIFL *gene expression in leaves of Zn-excess-treated plants (Figure 9): although six *OsZIFL *genes were expressed, only *OsZIFL7 *and *OsZIFL12 *were up-regulated, while all other family members did not change their expression levels. Considering the results obtained with Zn-excess and Fe-deficiency, it is possible to suggest that transcriptional regulation of most *OsZIFL *genes is more important in roots than in leaves, regardless of the level of expression in control conditions.

### *OsZIFL *duplicated pairs are co-expressed in specific plant organs and in response to stresses

**Figure 12:**
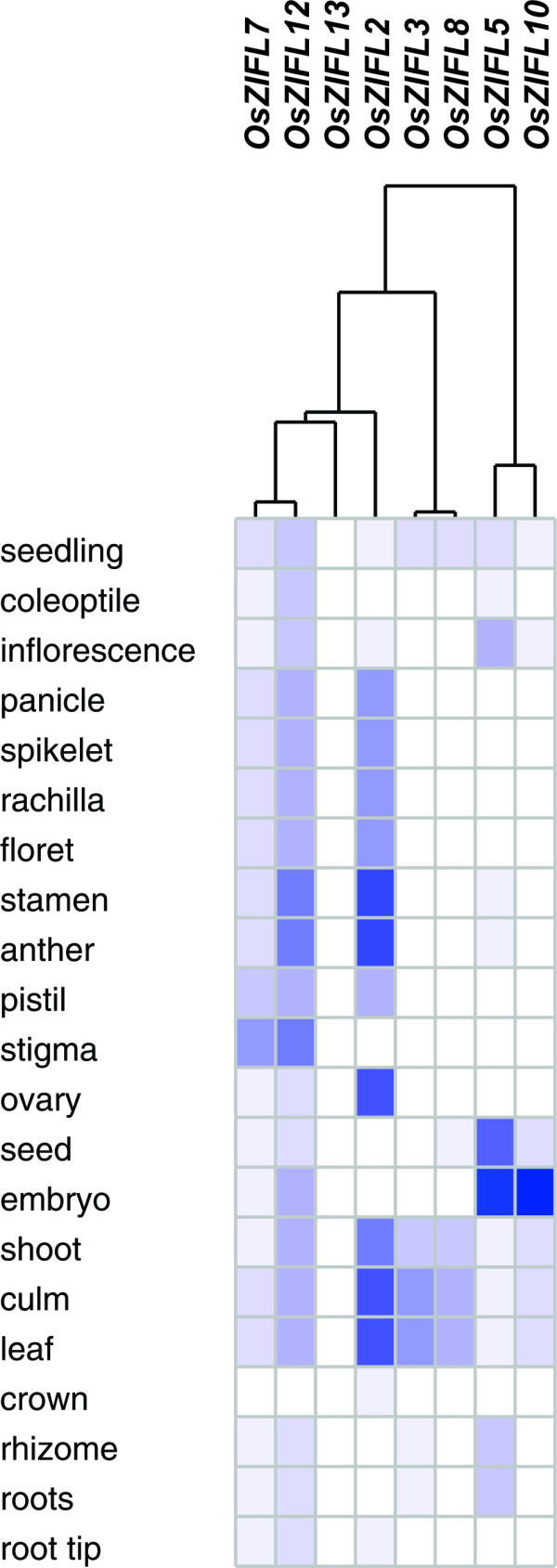
***OsZIFL2*, *OsZIFL3*, *OsZIFL5*, *OsZIFL7 OsZIFL10*, *OsZIFL12 *and *OsZIFL13 *gene expression data obtained using Genevestigator, and based on Affymetrix specific probes**. All available high quality arrays on rice organ-specific expression were used. All data from arrays showing expression under diverse treatments or from mutant/transgenic plants were kept out. Expression level is denoted by intensity of blue color. Organ names are given at left.

To analyze the expression pattern of *OsZIFL *genes based on microarray meta-analysis, we used Genevestigator [63]. Affymetrix unique probes used for expression analyses of *OsZIFL2*, *OsZIFL3*, *OsZIFL5*, *OsZIFL7*, *OsZIFL8*, *OsZIFL10*, *OsZIFL12 *and *OsZIFL13 *are listed in Additional File 7. The available data on expression of *OsZIFL *genes in different organs of rice plants is shown in Figure 12. Clearly, the expression pattern within each one of the duplicated gene pairs *OsZIFL5*-*OsZIFL10 *and *OsZIFL7*-*OsZIFL12 *cluster together, indicating their overlapping expression. According to microarray data, *OsZIFL5 *and *OsZIFL10 *are highly expressed in seed tissues, while *OsZIFL7 *and *OsZIFL12 *are expressed in reproductive organs and shoot tissues (Figure 12). Similarly, our qPCR experiments showed higher expression of both *OsZIFL7 *and *OsZIFL12 *in flag leaves and panicles and lower in roots (Figures 6G and 6H). The pair *OsZIFL8 *and *OsZIFL13*, which had no detected expression in our qPCR experiments, was evaluated using specific probes. While *OsZIFL13 *showed no expression, low expression of *OsZIFL8 *was observed in shoot tissues. Although qPCR will never generate the large amount of data that is achieved by cDNA microarrays, PCR has the advantage of unparalleled sensitivity, and therefore slight discrepancies are expected [64].

**Figure 13:**
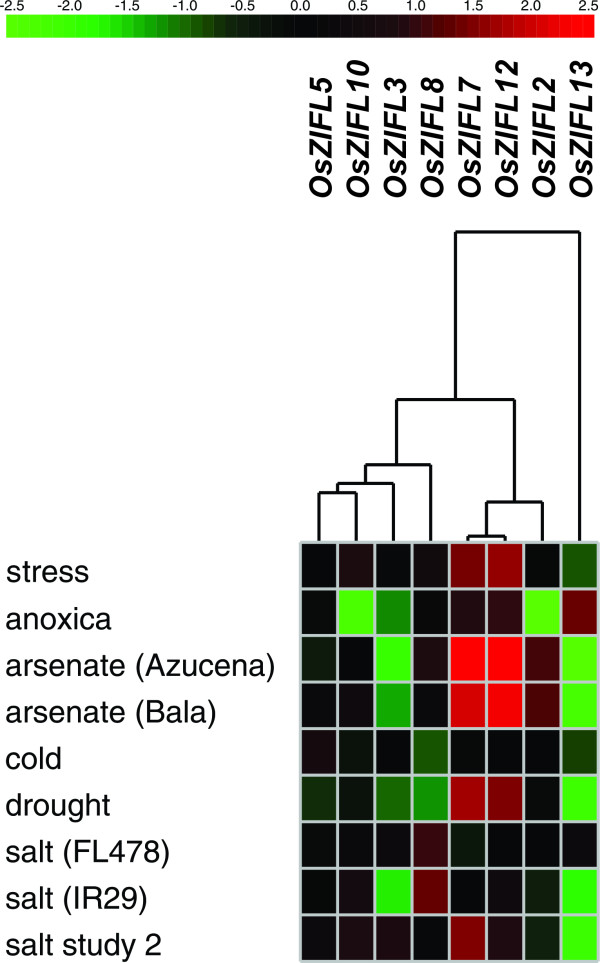
***OsZIFL2*, *OsZIFL3*, *OsZIFL5*, *OsZIFL7 OsZIFL10*, *OsZIFL12 *and *OsZIFL13 *gene expression data obtained using Genevestigator, and based on Affymetrix specific probes**. Only high quality arrays of rice expression under diverse treatments were used. Fold change in expression level is denoted by intensity of red color (for up-regulation) or green color (for down-regulation). Treatment names are given at left. The genotypes indicated between brackets (Azucena, Bala, FL478 and IR29) are considered, respectively, sensitive to arsenate, tolerant to arsenate, tolerant to salt and sensitive to salt.

*OsZIFL *duplicated pairs also show co-expression under stress conditions (Figure 13). *OsZIFL7 *and *OsZIFL12 *are highly up-regulated by arsenate in roots of an arsenate-tolerant (Bala) and an arsenate-sensitive (Azucena) cultivars (Figure 13) [65]. This suggests that these transporters could be responsive to general stress, as they are also up-regulated by Zn-excess and Fe-deficiency (Figures 8E and 8F; 9E and 9F, 10E and 10F). *OsZIFL2 *is also responsive to arsenate (Figure 13). *OsZIFL7 *and *OsZIFL12 *are also up-regulated under drought and salt stresses (Figure 13). *OsZIFL5 *and *OsZIFL10 *are mostly co-expressed, although no marked increase or decrease in expression was observed for both genes (Figure 13). The microarray results indicate a strong co-expression of the recently duplicated gene pairs *OsZIFL7-OsZIFL12 *and *OsZIFL5-OsZIFL10*, in accordance with our qPCR data.

## Discussion

### *ZIFL *expansion through segmental duplication

Phenotypic variation is not necessarily the result of entirely new genes. Instead, redundancy generated through gene duplication can be the source of evolutionary novelty. Plants are highly susceptible to duplication events, as most (if not all) have experienced whole-genome duplication events in their evolutionary past, as well as tandem and segmental duplications [66,67]. After duplication, gene copies can follow (1) neofunctionalization, where one copy maintains the ancestral function and the other can explore new evolutionary terrain; (2) pseudogenization, where one copy accumulates mutations and lose function while the other maintains the ancestral function; (3) subfunctionalization, in which deleterious mutations make one copy to be partially functional, but complementary to the other (i.e. in regard to the ancestral gene) [67,68]. As deleterious mutations are expected to be more common than beneficial ones, subfunctionalization is considered to be a more common fate for duplicated copies than neofunctionalization, and examples are already known [69,70]. These mutations are also more common in regulatory regions (i.e. promoters) than in functional motifs, where selective pressure is stronger; therefore, changes in expression patterns and/or changes in the responses to stimuli are probably more frequent [68].

In this work, we described the ZIFL protein family in plants, which is part of the MFS superfamily. We suggested that ZIFL proteins experienced an expansion in the monocot lineage, as we found three to four gene copies in dicots and eight up to thirteen in monocots, with all monocot paralogs grouping together (Figure 2). We further characterized the genomic organization of *ZIFL *genes in rice, and found that ten out of thirteen copies are located in a duplicated region of chromosomes 11 and 12 (Figure 3A). This region was first described as a recent segmental duplication, estimated from five to seven MYA [11,18]. This estimation was based on the high degree of similarity between terminal segments of both chromosomes (Figure 3). However, recent data showed that the duplication of this genomic segment is ancestral to the split of *S. bicolor*, *B. distachyon *and rice [14,19]. Wang et al. proposed that three rounds of unequal crossing-over events have produced the high similarity observed [9]. Thus, variation in sequence similarity within these regions reflects rather the antiquity of the unequal crossing-over events, than the date of segmental duplication as suggested earlier [11,18]. Gene conversion is also occurring at high frequencies within this region, further contributing for the maintenance of high similarity [9,13,15]. Using paralog pairs within the 3 Mb of chromosomes 11 and 12 from all species in the *Oryza *genus, a recent work demonstrated that concerted evolution is recurrent in this region for *Oryza *species [19]. Gene conversion was specifically found between *OsZIFL4 *and *OsZIFL9 *in *indica *rice, suggesting that concerted evolution has participated in the evolution of *ZIFL *genes [9].

We also demonstrated that the region where *S. bicolor ZIFL *genes are located in chromosome 8 is inverted in relation to its homologous region in rice chromosome 12 (Figures 3B and 3C). This inverted region was recently described for both *S. bicolor *and *B. distachyon*, encompassing 0.8 Mb [19]. *S. bicolor ZIFL *gene pairs are not as similar as rice paralogs, indicating that *S. bicolor ZIFL *genes probably did not undergo the same degree of concerted evolution as rice paralogs (i.e. unequal crossing-over and gene conversion). In agreement with that, Wang et al. used paralog quartets from rice and *S. bicolor *(i.e. a duplicated gene pair from rice and their homologs from *S. bicolor*) to search for gene conversions. They found that *OsZIFL4 *and *OsZIFL9 *went through whole gene conversion after the split between rice and *S. bicolor*, while *S. bicolor *homologs did not show conversion (in their supplemental table) [15]. Inversions are known to reduce the probability of recombination and to facilitate the maintenance of differences between interbreeding populations [71,72]. These results suggest that the inversion observed in *S. bicolor *reduced the probability of concerted evolution in the *SbZIFL *genes when compared to rice paralogs.

### Sequence and expression analyses suggest new functional sites in OsZIFL proteins and insights about duplicated gene pairs

Our analysis on motif composition of OsZIFL proteins also revealed interesting features of this family in rice. Together with the exon-intron organization (Figure 4), motif composition of duplicated genes *OsZIFL8 *and *OsZIFL13 *and their partial duplicated copy *OsZIFL3 *suggests that these genes are diverging in a higher rate when compared to other *OsZIFL *paralogs. They all show no ZIFL signature Cys motif C-P-G-C (Additional File 4). OsZIFL3 and OsZIFL8 also lack the MFS signature and OsZIFL13 lacks both MFS and antiporter signatures (Additional File 4). *OsZIFL3 *expression was detected in leaves, but at relatively low levels (Figure 6B). *OsZIFL8 *and *OsZIFL13 *transcripts were not detected in any of our qPCR experiments, and cDNAs corresponding to them are not present at the KOME database (http://cdna01.dna.affrc.go.jp/PIPE/). However, microarray metadata showed low expression of *OsZIFL8 *in shoots, although no expression of *OsZIFL13 *was detected in all plant organs evaluated (Figure 12). Further experiments should clarify if these genes are gaining new functions or accumulating mutations to become pseudogenes.

A variable region, which corresponds to a cytoplasmic loop, occupies a central position in the OsZIFL proteins (Figures 1B, 5A and Additional File 6). There is very low amino acid conservation within this loop. For this reason, we were able to find conserved motifs within the variable region only when using the whole ZIFL protein dataset in our analyses (Figure 5B). Variable regions are often found in transporters [50,51]. In the ZIP family (Zinc-regulated/Iron-regulated transporter Proteins), a variable region is considered to be the metal-binding site, as these loops are rich in histidine residues [50,51]. Our motif analysis in the ZIFL variable region detected some residues in conserved positions. In the CDF family, substrate specificity was proposed to be determined by few amino acids, normally histidine (H) or aspartic acid (D), which are, respectively, positively and negatively charged [52]. In OsZIFL proteins, lysine (K) and glutamic acid (E), also positive and negative residues, seem to be conserved in the variable loop (Figure 4B), although aspartic acid (D) and leucine (L) are also frequent (Figure 5B). This region and its conserved residues emerge as candidates for mutagenesis studies to clarify their importance in substrate transport, although no substrate was proven to be transported by ZIFL proteins [34]. Moreover, we described conserved motifs specific to ZIFL proteins (Figure 1B), which also contain candidate residues for site-directed mutagenesis studies.

We characterized the expression of *OsZIFL *genes in rice vegetative and reproductive organs (Figure 6) and compared the expression patterns of three duplicated gene pairs, *OsZIFL4*-*OsZIFL9*, *OsZIFL5*-*OsZIFL10 *and *OsZIFL7*-*OsZIFL12 *(Figure 6). *OsZIFL4 *and *OsZIFL9 *are both expressed in panicles at R7 stage, but only *OsZIFL4 *is expressed in roots (Figures 6C and 6D). This partial overlap suggests that their ancestral gene was at least expressed in panicles at R7 and in roots, as deleterious mutations could be subfunctionalizing *OsZIFL9 *(i.e. turning into a panicle-specific gene) while *OsZIFL4 *maintains both panicle and root expression. However, neofunctionalization of *OsZIFL4 *cannot be discarded. In agreement with that, Throude et al. showed that, from 115 duplicated gene pairs, the vast majority have been neofunctionalized or subfunctionalized, as 88%, 89% and 96% of duplicates, respectively expressed in grain, leaf and root, show distinct expression patterns [73]. A recent work in rice showed that the average number of conserved motifs between duplicated gene pairs declines with increased expression diversity, partially supporting the subfunctionalization model [74]. This is in accordance with the observed divergent expression of *OsZIFL4 *and *OsZIFL9 *and their motif composition, because OsZIFL9 has lost one N-terminal and two C-terminal motifs (Figure 5A). Expression patterns within the gene pairs *OsZIFL5*-*OsZIFL10 *and *OsZIFL7*-*OsZIFL12 *are similar (Figure 6), and both OsZIFL5 and OsZIFL10 have the same motif composition (Figure 5A). OsZIFL12 has two C-terminal motifs which are lacking in OsZIFL7 (Figure 5A), but expression of *OsZIFL7 *and *OsZIFL12 *is quite similar, as both are up-regulated in roots and leaves under Zn-excess (Figures 8E, 8F, 9E and 9F) and in roots under Fe-deficiency (Figures 10E and 10F). Microarray data also shows that the *OsZIFL *duplicated pairs *OsZIFL5*-*OsZIFL10 *and *OsZIFL7*-*OsZIFL12 *are co-expressed in the same plant organs and under the same treatments (Figures 12 and 13). Yim et al. showed that duplicated gene pairs with high local similarity (HLS) segments show higher expression correlations than gene pairs without these segments [74]. This probably results in an increased likelihood of gene conversion in promoters of gene pairs harboring HLS [74]. As gene conversion is known to homogenize sequences in multigene families, this probably explains the similar expression patterns of *OsZIFL *pairs, although it is established that duplicated gene pairs tend to rapidly diverge in their expression patterns [12,13,75].

### Expression of *OsZIFL *genes is involved in the partially overlapping pathways of Zn-excess and Fe-deficiency responses

Ten out of thirteen *OsZIFL *genes are found in two tandem groups of five genes in rice chromosomes 11 and 12, probably as a result of repeatedly tandem duplication events. This size of tandemly arrayed genes was estimated to be very rare, as only 7% of gene arrays in the rice genome have more than three genes [76]. Tandem duplication events have a tendency to be retained when involving genes for which fluctuation in copy number is unlikely to affect downstream genes, such as those at the end of or in flexible steps of pathways [76]. In *Arabidopsis *and rice, tandemly arrayed genes are enriched for membrane proteins and genes with function on abiotic and biotic stresses [76]. Moreover, tandemly arrayed genes often share regulatory *cis*-elements and tend to be expressed in a coordinated manner, as well as family members with HLS generated through gene conversion [74,77]. These observations are in accordance with the up-regulation of *OsZIFL *members under Zn-excess or Fe-deficiency, some of which show strong up-regulation upon stress imposition, mostly in roots (Figures 8, 9, 10 and 11). It also agrees with the enrichment observed for CATGC and IDE1-like elements in *OsZIFL *promoter sequences (Figure 7). Enrichment for the CATGC-box is related to Fe-deficiency responses in rice [30,78]. The rice specific gene *OsMIR *is strongly up-regulated by Fe-deficiency and shows 10 CATGC-boxes in its promoter sequence [78]. In another work, CATGC was shown to be enriched in promoters of genes regulated by OsIDEF1, an upstream transcription factor involved in the early response to Fe-deficiency [30]. Thus, *OsZIFL *genes which are responsive to Fe-deficiency are potentially under the same control network, although more data is necessary to confirm this hypothesis. Moreover, a similar up-regulation pattern is also observed in the *Arabidopsis AtZIF1 *gene, which is also responsive to both Zn-excess and Fe-deficiency [34,57,58]. This suggests that *OsZIFL *genes which are responsive to both stresses could have conserved regulatory sequences in comparison to *AtZIF1*.

Partial overlap between Zn-excess and Fe-deficiency response has been reported [59]. Zn-excess treated plants show much higher concentrations of Fe in roots, but slightly decreased Fe in shoots and inhibited expression of *OsFER1 *[59]. This indicates that Zn-excess causes Fe-deficiency due to mislocalization of the available Fe [59]. On the other hand, Fe-deficiency can cause Zn-excess, as Fe regulated transporters such as OsIRT1 are suggested to transport Zn and *Arabidopsis *plants under Fe-deficiency accumulate excessive Zn [39,79]. It was also demonstrated that 13.75% of the Zn-excess up-regulated genes in roots are also up-regulated by Fe-deficiency, further indicating an overlap between these stresses [59]. Excessive Zn was also shown to induce more genes in rice roots than in shoots, as 400 genes were induced in roots, while only 54 in shoots of *Arabidopsis *plants under Zn-excess [59].

OsIRO2, a bHLH (basic Helix-Loop-Helix) transcription factor induced by Fe-deficiency, is the regulator of Fe-deficiency responsive genes in roots, such as the genes *OsNAS1 *(*nicotianamine synthase 1*), *OsNAS2*, *OsNAAT1 *(*nicotianamine amino-transferase 1*), *OsDMAS1 *(*deoxymugineic acid synthase 1*) and the DMA-Fe^3+ ^transporter *OsYSL15 *[80]. Expression of *OsIRO2 *was shown to be up-regulated by and to control the induction of these genes under Zn-excess [59]. However, *OsIRT1*, a classical Fe-deficiency-regulated gene, is not regulated by OsIRO2 [80], and is not up-regulated under Zn-excess [59]. These results indicate that OsIRO2 is in the crosslink between Zn-excess and Fe-deficiency responses. The OsIRO2 binding site CACGTGG is not found in *OsZIFL *promoters, but our qPCR data shows that *OsZIFL4*, *OsZIFL5*, *OsZIFL7 *and *OsZIFL12 *are up-regulated in roots by both stresses (Figures 8 and 10). Considering our results, it is possible to suggest that *OsZIFL *genes are part of the overlapping pathway that links Fe-deficiency and Zn-excess, although regulators different from OsIRO2 may control their expression. One of these regulators could be IDEF1 [54].

## Conclusions

As the first description of the *ZIFL *family in plants, this work is the basis for functional studies, especially in rice. We have shed light onto the unusual genomic distribution of *OsZIFL *genes, and made suggestions about the evolutionary forces that shaped the high degree of similarity between them. We also characterized in detail the motif composition of rice *OsZIFL *genes and the expression patterns in different rice organs and under stress conditions. More functional data, such as loss-of-function mutants, sub cellular localization and ligand specificity, are necessary to uncover the specific roles of each protein and to know to what extent they are functionally redundant, as well as to clarify the roles of *OsZIFL *genes in the homeostasis of Zn and Fe in rice.

## Methods

### Plant material and treatments

Rice seeds of the Nipponbare cultivar were germinated for four days in petri dishes, soaked in distilled water at 28°C (two days in the dark, two days in the light). After germination, seedlings were transferred to holders positioned over plastic pots with five liters of nutrient solution (16 seedlings per pot) containing 700 μM K_2_SO_4_, 100 μM KCl, 100 μM KH_2_PO_4_, 2 mM Ca(NO_3_)_2_, 500 μM MgSO_4_, 10 μM H_3_BO_3_, 0.5 μM MnSO_4_, 0.5 μM ZnSO_4_, 0.2 μM CuSO_4_, 0.01 μM (NH_4_)_6_Mo_7_O_24_, and 100 μM Fe^+3^-EDTA. The pH of the nutrient solution was adjusted to 5.4. Plants were kept at 28°C ± 1°C under photoperiod of 16h/8h light/dark (150 μmol.m^-2^.s^-1^). Solutions were replaced every 3-4 days.

For expression analyses in vegetative organs, samples of roots, leaves and culms were collected at the four-leaf stage (approximately 30 days of growth). For expression analyses in reproductive organs, plants were grown in soil under flooded conditions in an experimental unit at IRGA (Instituto Rio-Grandense do Arroz), in Cachoeirinha, RS, Brazil (29°54'58.61''S 51°10'02.65''W), during the rice growing season (October 2007 to March 2008). Soil characteristics of this site were reported by Stein et al. [36]. Samples of flag-leaves and panicles were collected during R3 (panicle exertion), R5 (grain filling) and R7 (grain dry-down) stages, according to Counce et al. [81]. Laboratory grown plants at the four-leaf stage were submitted to Zn-excess or to Fe-deficiency. For Zn-excess, plants were kept in 200 μM of ZnSO_4 _for 3 days. For Fe-deficiency, Fe^+3^-EDTA was omitted from nutrient solution and samples were collected after 7 days. In all experiments, three biological samples composed of at least three plants each were used for gene expression analyses.

### Sequence retrieval and databases

Sequences of *Arabidopsis thaliana *AtZIF1 (AT5G13740), AtZIFL1 (AT5G13750) and AtZIFL2 (AT3G43790) proteins were downloaded from the TAIR database (The *Arabidopsis *Information Resource, http://www.arabidopsis.org/) and used as queries to search the rice genome at the TIGR Rice Genome database (http://rice.plantbiology.msu.edu/) for *ZIFL *sequences using tBLASTn and BLASTp. Sequences with an expected value lower than 1 × 10^-30 ^and harboring more than 30% of similarity considering 30% of the sequence were selected. Both *Arabidopsis *and rice sequences were then used as queries to survey the genomes of *Vitis vinifera*, *Populus trichocarpa*, *Sorghum bicolor*, *Brachypodium distachyon*, *Zea mays*, *Selaginella moellendorffii *and *Physcomitrella patens *at the Phytozome database (http://www.phytozome.net/), using the same criteria as above. All plant sequences found (plus the previously known *Arabidopsis *sequences) were aligned and used as an input to build an HMM profile using the HMMER package [82]. The ZIFL HMM profile consensus sequence was used to re-search the listed genomes. As new sequences were found, the procedure was repeated iteratively until no new sequence appeared. To visualize the ZIFL HMM profile and the conserved motifs we used LogoMat M [83]. Alignments of ZIFL profile to MFS_1 HMM profile were performed using LogoMat P [84]. Individual sequences were manually curated to discard those of poor quality or incomplete (not starting with methionine or not having a stop codon). Accession numbers, given nomenclature, chromosome and genomic positions and predicted number of transmembrane domains (TMs) of ZIF-like proteins are shown in Additional File 1. Two unnanotated *ZIFL *genes from *Zea mays *were predicted using Fgenesh (http://www.softberry.com) and their given nomenclature, chromosome and genomic positions, exon coordinates and predicted number of TMs are shown in Additional File 2.

Sequences from MFS_1 proteins from each monocot and dicot species analyzed in this work were retrieved using the same method as for ZIFL protein sequences. The consensus sequence generated from the HMM profile of MFS_1 (Pfam number PF07690) was used to search the genomes. All locus numbers of *MFS_1 *genes used are given in Additional File 8.

### Alignments and phylogenetic analyses

Sequence alignment and phylogenetic analyses for ZIFL proteins were performed using the MEGA (Molecular Evolutionary Genetics Analysis) 4.1 package [85]. Protein multiple alignments were obtained with ClustalW and phylogenetic trees were reconstructed with the neighbor-joining method and the following parameteres: pairwise deletion option, 1,000 replicates of bootstrap and Poisson correction distance. The consensus tree shows only branches with a bootstrap consensus >50. Bayesian analysis was applied to generate a posterior probability distribution using the Metropolis-coupled Markov Chain Monte Carlo (MCMC) with MrBayes 3.0b4 [86,87]. The search was run for 1 × 10^6 ^generations, and every 100th tree was sampled. Posterior probabilities for each branch were calculated from the sampled trees. Sequence alignments of MFS_1 proteins were performed using MEGA 4.1 package, and phylogenetic trees were reconstructed with the neighbor-joining method, following the same parameters described above.

For genomic alignments, we used a 100 kb region from rice chromosomes 11 and 12 and *S. bicolor *chromosomes 5 and 8 spanning the tandemly repeated *ZIFL *genes regions in both species. The graphic alignment tool for comparative sequence analysis (GATA) was used to align the sequences and visualize the results [88]. GATA uses BLASTn to compare a reference to a comparative sequence. A sliding window of predefined size slides through the reference sequence and aligns it to the comparative sequence. Matches are shown in black for forward hits (+/+) and in red for reverse hits (+/-). We used a window size of 24 and a lower cutoff score of 80. The default values were used for the other settings.

### Exon-intron determination, motif finding and promoter analysis

For determining exon-intron organization, genomic and coding sequences (predicted, cDNA when available) were aligned. To search for conserved motifs in ZIF-like proteins, MEME (Motif EM for Motif Elicitation - http://meme.nbcr.net/[49]) was used, with the following parameters: zero or one motif per sequence, 6 and 300 amino acids as minimum and maximum sizes of motifs. Only motifs with expected value lower than 1 × 10^-20 ^were considered. For motifs within the variable region, the e-value cutoff was increased to 1X 10^-10 ^due to high sequence divergence. The best possible match of each motif was searched in the InterPro database (http://www.ebi.ac.uk/interpro/). To identify the transmembrane domains of ZIFL proteins we used Conpred II (http://bioinfo.si.hirosaki-u.ac.jp/~ConPred2/), a consensus prediction method for obtaining transmembrane topology models. Promoter sequences from -1,500 bp to +1 bp of each rice *ZIF-like *gene were extracted from the TIGR Rice Genome database. Different strategies were used to find regulatory sequences within the promoters of *OsZIF-like *genes. POCO was used to compare the promoter dataset to the *Arabidopsis thaliana *clean background, the closest species available for this tool [53]. POCO was run with default settings, except for the pattern length selected as 5 bp. To confirm that over-represented motifs in comparison to *Arabidopsis *background are also over-represented when compared to rice background, the -1,500 bp to +1 bp promoter region of nearly 25,000 rice genes were downloaded from the Osiris database (http://www.bioinformatics2.wsu.edu/cgi-bin/Osiris/cgi/home.pl/[55]) and evaluated for average number of motifs.

### Genevestigator

We used only specific Affymetrix probes for rice *ZIF *genes (Additional File 7) to analyze expression data from GENEVESTIGATOR (http://www.genevestigator.com) [63]. Only high quality arrays were used.

### RNA extraction and cDNA synthesis

Rice tissues were harvested from plants grown under laboratory or field conditions as described above. Total RNA was extracted using the Concert Plant RNA Reagent (Invitrogen^®^, Carlsbad, CA, USA) and treated with DNase I (Invitrogen^®^, Carlsbad, CA, USA). cDNA was prepared using the SMART PCR cDNA Synthesis Kit by Clontech^® ^Laboratories (Mountain View, CA, USA), according to the manufacturer's instructions. First-strand cDNA synthesis was performed with oligo dT and reverse transcriptase (M-MLV, Invitrogen^®^, Carlsbad, CA, USA) using 1 μg of RNA.

### Quantitative RT-PCR and data analysis

For quantitative RT-PCR analysis (qPCR), the synthesized first strand cDNA from each time point was diluted 100 times. qPCR was carried out in an Applied Biosystems StepOne real-time cycler. All primers (listed in Additional File 9) were designed to amplify 100-150 bp of the 3'-UTR of the genes and to have similar Tm values (60 ± 2°C). Reaction settings were composed of an initial denaturation step of 5 min at 94°C, followed by 40 cycles of 10 s at 94°C, 15 s at 60°C, 15 s at 72°C; samples were held for 2 min at 60°C for annealing of the amplified products and then heated from 60 to 99°C with a ramp of 0.3°C/s to provide the denaturing curve of the amplified products. qPCRs were carried out in 20 μl final volume composed of 10 μl of each reverse transcription sample diluted 100 times, 2 μl of 10X PCR buffer, 1.2 μl of 50 mM MgCl_2_, 0.1 μl of 5 mM dNTPs, 0.4 μl of 10 μM primer pairs, 4.25 μl of water, 2.0 μl of SYBR green (1:10,000, Molecular Probe), and 0.05 μl of Platinum Taq DNA polymerase (5 U/μl, Invitrogen^®^, Carlsbad, CA, USA). Obtained data were analyzed using the comparative C_t _(threshold cycle) method [89]. The PCR efficiency from the exponential phase (E) was calculated for each individual amplification plot using the LinRegPCR software [90]. In each plate, the average of PCR efficiency for each amplicon was determined and used in further calculations. C_t _values for all genes were normalized to the C_t _value of *UBQ5 *[91]. The equation Q_0 target gene_/Q_0 UBQ5 _= [(Eff _UBQ5_) ^Ct UBQ5^/(Eff _target gene_) ^Ct target gene^], where Q_0 _corresponds to the initial amount of transcripts, was used for normalization [89]. Each data point corresponds to three true biological replicate samples.

### Statistical analyses

When appropriate, data were subjected to ANOVA and means were compared by the Tukey HSD or Student's *t *test using the SPSS Base 12.0 for Windows (SPSS Inc., USA).

## Abreviations

CDF: cation diffusion facilitator; CDS: coding sequence; OsDMAS1: deoxymugineic acid synthase; DDC: duplication-degeneration-complementation; HLS: high local similarity; IDE1: iron-deficiency responsive element 1; IDEF1: iron-deficiency responsive element-binding factor 1; MFS: major facilitator superfamily; MYA: million years ago; NAAT1: nicotianamine amino transferase; NAS: nicotianamine synthase; ORF: open reading frame; ZIF: zinc-induced facilitator; ZIFL: zinc-induced facilitator-like; ZIP: zinc-regulated/iron-regulated transporter protein

## Authors' contributions

FKR designed the experiments and drafted the manuscript. FKR performed the alignments, phylogenetic, motif composition, promoter and microarray expression analyses, and participated in qPCR experiments. RAS, PKM, ERS and KLL performed qPCR experiments. FKR and RAS analyzed qPCR data. RAS helped drafting the manuscript. JPF conceived and coordinated the study and prepared the final manuscript. All authors read and approved the final manuscript.

## Supplementary Material

Additional File 1 ***ZIFL *gene sequence information**. Gene locus number, given name, chromosome number, genomic localization, strand, predicted coding sequence (CDS) and protein length, and predicted number of transmembrane domains (TM) are shown for each gene. Click here for file

Additional File 2 **Previously unannotated *ZIFL *genes**. Gene locus number, given name, chromosome number, genomic localization, strand, predicted coding sequence (CDS) and protein length, exon positions and predicted number of transmembrane domains (TM) are shown for each gene. Click here for file

Additional File 3 **Phylogenetic trees of ZIFL and MFS_1 proteins**. Phylogenetic trees showing the separation of ZIFL proteins from the other MFS_1 sequences in each monocot and dicot species analyzed. (A) *Oryza sativa*, (B) *Sorghum bicolor*, (C) *Zea mays*, (D) *Brachypodium distachyon*, (E) *Arabidopsis thaliana*, (F) *Vitis vinifera*, (G) *Populus trichocarpa*. Click here for file

Additional File 4 **Conserved residues found in ZIFL protein sequences**. Residues of cysteine (Cys) motif, histidine (His) motif, TM8-TM9 loop motif, and residues of MFS and antiporter signatures of each ZIFL protein are shown. Click here for file

Additional File 5 **Chromosomal positions of *ZIFL *genes**. Chromosomal positions of *ZIFL *genes in (A) *Oryza sativa*, *Sorghum bicolor *and *Brachypodium distachyon *chromosomes, and in (B) *Zea mays *chromosomes. Only *ZIFL*-containing chromosomes are shown. Non-*ZIFL *genes within *ZIFL *gene clusters were omitted. Click here for file

Additional File 6 **Alignment of OsZIFL protein sequences**. Alignment was constructed using ClustalW. Conserved amino acids are marked in black or grayscale according to similarity level. Click here for file

Additional File 7 **Probes used in Genevestigator analyses and evaluation of specificity of rice ZIFL Affymetrix**^® ^**microarray probes**. For each *ZIFL *gene locus number, all corresponding probes are listed. Each probe is classified as unique or not unique, and, in the second case, the number of the other locus matching to the probe is provided. Click here for file

Additional File 8 **Locus numbers of *MFS_1 *genes used in reconstruction of phylogenetic trees, in addition to *ZIFL *genes**. Locus numbers of *MFS_1 *genes used in the reconstruction of phylogenetic trees shown in Additional File 3, in addition to *ZIFL *genes (Additional File 1 and Additional File 2). Click here for file

Additional File 9 **Gene-specific primers used for quantitative RT-PCR**. Sequences of PCR primers used in quantitative RT-PCR analyses of rice *ZIFL *gene expression. Click here for file
