# Supplementary material for: ZINC-INDUCED FACILITATOR-LIKE family in plants: lineage-specific expansion in monocotyledons and conserved genomic and expression features among rice (Oryza sativa) paralogs
Source: BMC Plant Biol. 2011 Jan 25;11:20. doi: 10.1186/1471-2229-11-20 (PMC3041735; doi:10.1186/1471-2229-11-20)
Supplement: Additional File 1 — ZIFL gene sequence information. Gene locus number, given name, chromosome number, genomic localization, strand, predicted coding sequence (CDS) and protein length, and predicted number of transmembrane domains (TM) are shown for each gene. [file 1471-2229-11-20-S1.DOC]

**Additional File 1.** *ZIFL* gene sequence information.

| **Locus number** | **Gene name** | **Chromosome number** | **Genomic localization** | **Strand** | **CDS size (base pairs)** | **Protein size (amino acids)** | **TM domains** |
| --- | --- | --- | --- | --- | --- | --- | --- |
|  |  |  |  |  |  |  |  |
| *Zea mays* |  |  |  |  |  |  |  |
| [GRMZM2G141081](http://www.phytozome.net/genePage.php?search=1&detail=1&crown&method=0&searchText=transcriptid%3A16930131) | *ZmZIFL2* | 2 | 145779171 - 145783123 | + | 1,479 | 492 | 12 |
| [GRMZM2G115658](http://www.phytozome.net/genePage.php?search=1&detail=1&crown&method=0&searchText=transcriptid%3A16924600) | *ZmZIFL3* | 2 | 145861576 - 145865621 | - | 1,509 | 502 | 12 |
| [GRMZM2G029219](http://www.phytozome.net/genePage.php?search=1&detail=1&crown&method=0&searchText=transcriptid%3A16905590) | *ZmZIFL4* | 3 | 42872091 - 42879804 | + | 1,473 | 490 | 12 |
| [GRMZM2G161310](http://www.phytozome.net/genePage.php?search=1&detail=1&crown&method=0&searchText=transcriptid%3A16934500) | *ZmZIFL5* | 3 | 45160852 - 45175101 | - | 1,347 | 448 | 12 |
| [GRMZM2G075594](http://www.phytozome.net/genePage.php?search=1&detail=1&crown&method=0&searchText=transcriptid%3A16915818) | *ZmZIFL6* | 3 | 228297411 - 228302606 | - | 1,437 | 478 | 11 |
| [GRMZM2G022375](http://www.phytozome.net/genePage.php?search=1&detail=1&crown&method=0&searchText=transcriptid%3A16904120) | *ZmZIFL7* | 4 | 185559013 - 185562973 | + | 1,497 | 498 | 12 |
| [GRMZM2G456923](http://www.phytozome.net/genePage.php?search=1&detail=1&crown&method=0&searchText=transcriptid%3A16948177) | *ZmZIFL9* | 8 | 1809029 - 1814823 | + | 1,443 | 480 | 11 |
| [GRMZM2G311401](http://www.phytozome.net/genePage.php?search=1&detail=1&crown&method=0&searchText=transcriptid%3A16921517) | *ZmZIFL10* | 10 | 3684222 - 3687765 | - | 1,278 | 425 | 10 |
|  |  |  |  |  |  |  |  |
| *Oryza sativa* |  |  |  |  |  |  |  |
| [LOC_Os01g16260](http://www.phytozome.net/genePage.php?search=1&detail=1&crown&method=0&searchText=transcriptid%3A16831123) | *OsZIFL1* | 1 | 9202963 - 9210788 | - | 1,461 | 486 | 12 |
| [LOC_Os01g17214](http://www.phytozome.net/genePage.php?search=1&detail=1&crown&method=0&searchText=transcriptid%3A16831236) | *OsZIFL2* | 1 | 9888524 - 9901714 | - | 1,494 | 497 | 12 |
| [LOC_Os07g08300](http://www.phytozome.net/genePage.php?search=1&detail=1&crown&method=0&searchText=transcriptid%3A16874221) | *OsZIFL3* | 7 | 4239045 - 4252498 | + | 1,071 | 356 | 8 |
| [LOC_Os11g04020](http://www.phytozome.net/genePage.php?search=1&detail=1&crown&method=0&searchText=transcriptid%3A16887753) | *OsZIFL4* | 11 | 1617495 - 1622979 | - | 1,422 | 473 | 11 |
| [LOC_Os11g04030](http://www.phytozome.net/genePage.php?search=1&detail=1&crown&method=0&searchText=transcriptid%3A16887754) | *OsZIFL5* | 11 | 1625475 - 1630422 | - | 1,413 | 470 | 11 |
| [LOC_Os11g04060](http://www.phytozome.net/genePage.php?search=1&detail=1&crown&method=0&searchText=transcriptid%3A16887760) | *OsZIFL6* | 11 | 1640193 - 1644545 | - | 1,329 | 442 | 10 |
| [LOC_Os11g04104](http://www.phytozome.net/genePage.php?search=1&detail=1&crown&method=0&searchText=transcriptid%3A16892118) | *OsZIFL7* | 11 | 1656864 - 1667363 | - | 1,515 | 504 | 11 |
| [LOC_Os11g04150](http://www.phytozome.net/genePage.php?search=1&detail=1&crown&method=0&searchText=transcriptid%3A16887769) | *OsZIFL8* | 11 | 1680094 - 1687100 | - | 1,197 | 398 | 9 |
| [LOC_Os12g03830](http://www.phytozome.net/genePage.php?search=1&detail=1&crown&method=0&searchText=transcriptid%3A16892540) | *OsZIFL9* | 12 | 1567001 - 1571324 | - | 1,233 | 410 | 10 |
| [LOC_Os12g03860](http://www.phytozome.net/genePage.php?search=1&detail=1&crown&method=0&searchText=transcriptid%3A16892543) | *OsZIFL10* | 12 | 1583171 - 1588135 | - | 1,413 | 470 | 11 |
| [LOC_Os12g03870](http://www.phytozome.net/genePage.php?search=1&detail=1&crown&method=0&searchText=transcriptid%3A16892550) | *OsZIFL11* | 12 | 1593733 - 1599962 | - | 1,398 | 465 | 10 |
| [LOC_Os12g03899](http://www.phytozome.net/genePage.php?search=1&detail=1&crown&method=0&searchText=transcriptid%3A16892554) | *OsZIFL12* | 12 | 1605073 - 1615757 | - | 1,509 | 502 | 11 |
| [LOC_Os12g03950](http://www.phytozome.net/genePage.php?search=1&detail=1&crown&method=0&searchText=transcriptid%3A16892559) | *OsZIFL13* | 12 | 1634122 - 1641673 | - | 870 | 289 | 7 |
|  |  |  |  |  |  |  |  |
| *Sorghum bicolor* |  |  |  |  |  |  |  |
| [Sb03g010620](http://www.phytozome.net/genePage.php?search=1&detail=1&crown&method=0&searchText=transcriptid%3A1961304) | *SbZIFL1* | 3 | 11533503 - 11542093 | - | 1,476 | 491 | 11 |
| [Sb03g011240](http://www.phytozome.net/genePage.php?search=1&detail=1&crown&method=0&searchText=transcriptid%3A1961377) | *SbZIFL2* | 3 | 12431599 - 12437758 | + | 1,470 | 489 | 12 |
| [Sb03g011330](http://www.phytozome.net/genePage.php?search=1&detail=1&crown&method=0&searchText=transcriptid%3A1961389) | *SbZIFL3* | 3 | 12561627 - 12567442 | - | 1,686 | 561 | 11 |
| [Sb03g011340](http://www.phytozome.net/genePage.php?search=1&detail=1&crown&method=0&searchText=transcriptid%3A1961391) | *SbZIFL4* | 3 | 12581328 - 12586288 | - | 1,272 | 423 | 7 |
| [Sb03g011360](http://www.phytozome.net/genePage.php?search=1&detail=1&crown&method=0&searchText=transcriptid%3A1961395) | *SbZIFL5* | 3 | 12629659 - 12635989 | - | 1,467 | 488 | 12 |
| [Sb03g011490](http://www.phytozome.net/genePage.php?search=1&detail=1&crown&method=0&searchText=transcriptid%3A1961413) | *SbZIFL6* | 3 | 12823785 - 12828939 | - | 1,368 | 455 | 10 |
| [Sb03g023750](http://www.phytozome.net/genePage.php?search=1&detail=1&crown&method=0&searchText=transcriptid%3A1962017) | *SbZIFL7* | 3 | 47387326 - 47392154 | + | 1,350 | 449 | 9 |
| [Sb05g002050](http://www.phytozome.net/genePage.php?search=1&detail=1&crown&method=0&searchText=transcriptid%3A1969051) | *SbZIFL8* | 5 | 2228281 - 2232726 | - | 1,029 | 342 | 9 |
| [Sb05g002060](http://www.phytozome.net/genePage.php?search=1&detail=1&crown&method=0&searchText=transcriptid%3A1969052) | *SbZIFL9* | 5 | 2242553 - 2250395 | - | 1,500 | 499 | 11 |
| [Sb05g002070](http://www.phytozome.net/genePage.php?search=1&detail=1&crown&method=0&searchText=transcriptid%3A1969054) | *SbZIFL10* | 5 | 2259685 - 2265441 | - | 1,131 | 376 | 9 |
| [Sb05g008475](http://www.phytozome.net/genePage.php?search=1&detail=1&crown&method=0&searchText=transcriptid%3A1969903) | *SbZIFL11* | 5 | 15921143 - 15931827 | + | 1,407 | 469 | 12 |
| [Sb08g001400](http://www.phytozome.net/genePage.php?search=1&detail=1&crown&method=0&searchText=transcriptid%3A1977265) | *SbZIFL12* | 8 | 1360335 - 1374427 | + | 1,440 | 479 | 11 |
| [Sb08g001410](http://www.phytozome.net/genePage.php?search=1&detail=1&crown&method=0&searchText=transcriptid%3A1977268) | *SbZIFL13* | 8 | 1377818 - 1382667 | + | 1,209 | 402 | 9 |
| [Sb08g008410](http://www.phytozome.net/genePage.php?search=1&detail=1&crown&method=0&searchText=transcriptid%3A1978062) | *SbZIFL14* | 8 | 16290761 - 16298043 | + | 1,257 | 418 | 8 |
|  |  |  |  |  |  |  |  |
| *Brachypodium distachyon* |  |  |  |  |  |  |  |
| [Bradi2g10020](http://www.phytozome.net/genePage.php?search=1&detail=1&crown&method=0&searchText=transcriptid%3A16482037) | *BdZIFL1* | 2 | 8198619 - 8208021 | - | 1,734 | 577 | 11 |
| [Bradi2g10030](http://www.phytozome.net/genePage.php?search=1&detail=1&crown&method=0&searchText=transcriptid%3A16482038) | *BdZIFL2* | 2 | 8213555 - 8223919 | - | 1,353 | 450 | 11 |
| [Bradi2g10800](http://www.phytozome.net/genePage.php?search=1&detail=1&crown&method=0&searchText=transcriptid%3A16482129) | *BdZIFL3* | 2 | 9014441 - 9023865 | - | 1,497 | 498 | 12 |
| [Bradi4g26340](http://www.phytozome.net/genePage.php?search=1&detail=1&crown&method=0&searchText=transcriptid%3A16498224) | *BdZIFL4* | 4 | 31572862 - 31577612 | + | 1,305 | 434 | 9 |
| [Bradi4g26350](http://www.phytozome.net/genePage.php?search=1&detail=1&crown&method=0&searchText=transcriptid%3A16498225) | *BdZIFL5* | 4 | 31579009 - 31581798 | + | 1,026 | 341 | 7 |
| [Bradi4g26370](http://www.phytozome.net/genePage.php?search=1&detail=1&crown&method=0&searchText=transcriptid%3A16498228) | *BdZIFL6* | 4 | 31586269 - 31597410 | + | 1,485 | 494 | 11 |
| [Bradi4g26380](http://www.phytozome.net/genePage.php?search=1&detail=1&crown&method=0&searchText=transcriptid%3A16498232) | *BdZIFL7* | 4 | 31598606 - 31604405 | + | 1,494 | 497 | 10 |
| [Bradi4g43580](http://www.phytozome.net/genePage.php?search=1&detail=1&crown&method=0&searchText=transcriptid%3A16500363) | *BdZIFL8* | 4 | 47240624 - 47245218 | - | 1,527 | 508 | 12 |
| [Bradi4g43590](http://www.phytozome.net/genePage.php?search=1&detail=1&crown&method=0&searchText=transcriptid%3A16500365) | *BdZIFL9* | 4 | 47246889 - 47252698 | - | 1,479 | 492 | 11 |
| [Bradi4g43620](http://www.phytozome.net/genePage.php?search=1&detail=1&crown&method=0&searchText=transcriptid%3A16500370) | *BdZIFL10* | 4 | 47259935 - 47265014 | - | 1,374 | 457 | 11 |
|  |  |  |  |  |  |  |  |
| *Arabidopsis thaliana* |  |  |  |  |  |  |  |
| [AT5G13740](http://www.phytozome.net/genePage.php?search=1&detail=1&crown&method=0&searchText=transcriptid%3A17373643) | AtZIF1^a^ | 5 | 4432306 - 4436668 | + | 1,461 | 486 | 11 |
| [AT5G13750](http://www.phytozome.net/genePage.php?search=1&detail=1&crown&method=0&searchText=transcriptid%3A17373644) | AtZIFL1^a^ | 5 | 4438200 - 4441579 | + | 1,437 | 478 | 10 |
| [AT3G43790](http://www.phytozome.net/genePage.php?search=1&detail=1&crown&method=0&searchText=transcriptid%3A17364550) | AtZIFL2^a^ | 3 | 15655384 - 15660086 | + | 1,455 | 484 | 11 |
|  |  |  |  |  |  |  |  |
| *Populus trichocarpa* |  |  |  |  |  |  |  |
| [POPTR_0006s02710](http://www.phytozome.net/genePage.php?search=1&detail=1&crown&method=0&searchText=transcriptid%3A17342279) | *PtZIFL1* | scaffold_6 | 1722558 - 1728279 | + | 1,437 | 478 | 10 |
| [POPTR_0008s01110](http://www.phytozome.net/genePage.php?search=1&detail=1&crown&method=0&searchText=transcriptid%3A17316236) | *PtZIFL2* | scaffold_8 | 532910 - 538315 | + | 1,488 | 495 | 12 |
| [POPTR_0009s02680](http://www.phytozome.net/genePage.php?search=1&detail=1&crown&method=0&searchText=transcriptid%3A17332056) | *PtZIFL3* | scaffold_9 | 3436203 - 3443562 | - | 1,497 | 498 | 10 |
| [POPTR_0016s02500](http://www.phytozome.net/genePage.php?search=1&detail=1&crown&method=0&searchText=transcriptid%3A17325150) | *PtZIFL4* | scaffold_16 | 1231196 - 1238604 | + | 1,440 | 479 | 11 |
|  |  |  |  |  |  |  |  |
| *Vitis vinifera* |  |  |  |  |  |  |  |
| [GSVIVT00023334001](http://www.phytozome.net/genePage.php?search=1&detail=1&crown&method=0&searchText=transcriptid%3A1724285) | *VvZIFL1* | 8 | 10337395 - 10355258 | - | 1,431 | 476 | 11 |
| [GSVIVT00033966001](http://www.phytozome.net/genePage.php?search=1&detail=1&crown&method=0&searchText=transcriptid%3A1734917) | *VvZIFL2* | Un_random | 126574785 - 126599999 | + | 1,413 | 470 | 11 |
| [GSVIVT00033971001](http://www.phytozome.net/genePage.php?search=1&detail=1&crown&method=0&searchText=transcriptid%3A1734922) | *VvZIFL3* | Un_random | 126641091 - 126674624 | + | 1,467 | 488 | 11 |
| [GSVIVT00033972001](http://www.phytozome.net/genePage.php?search=1&detail=1&crown&method=0&searchText=transcriptid%3A1734923) | *VvZIFL4* | Un_random | 126681308 - 126695478 | - | 1,647 | 548 | 11 |
| [GSVIVT00033975001](http://www.phytozome.net/genePage.php?search=1&detail=1&crown&method=0&searchText=transcriptid%3A1734926) | *VvZIFL5* | Un_random | 126731215 - 126739779 | - | 1,464 | 487 | 10 |
|  |  |  |  |  |  |  |  |
| *Selaginella moellendorffii* |  |  |  |  |  |  |  |
| [164091](http://www.phytozome.net/genePage.php?search=1&detail=1&crown&method=0&searchText=transcriptid%3A15407448) | *SmZIFL1* | scaffold_0 | 4058805 - 4064564 | + | 1,485 | 494 | 11 |
| [408219](http://www.phytozome.net/genePage.php?search=1&detail=1&crown&method=0&searchText=transcriptid%3A15420283) | *SmZIFL2* | scaffold_8 | 1448426 - 1450923 | - | 1,497 | 498 | 11 |
| [147893](http://www.phytozome.net/genePage.php?search=1&detail=1&crown&method=0&searchText=transcriptid%3A15407953) | *SmZIFL3* | scaffold_17 | 1505890 - 1508475 | + | 1,545 | 514 | 11 |
| [96275](http://www.phytozome.net/genePage.php?search=1&detail=1&crown&method=0&searchText=transcriptid%3A15406286) | *SmZIFL4* | scaffold_18 | 585495 - 587800 | - | 1,362 | 453 | 10 |
| [412524](http://www.phytozome.net/genePage.php?search=1&detail=1&crown&method=0&searchText=transcriptid%3A15412271) | *SmZIFL5* | scaffold_18 | 589633 - 592168 | + | 1,353 | 450 | 10 |
| [157948](http://www.phytozome.net/genePage.php?search=1&detail=1&crown&method=0&searchText=transcriptid%3A15415971) | *SmZIFL6* | scaffold_73 | 557939 - 560436 | - | 1,461 | 486 | 9 |
| [184037](http://www.phytozome.net/genePage.php?search=1&detail=1&crown&method=0&searchText=transcriptid%3A15421729) | *SmZIFL7* | scaffold_90 | 208963 - 214635 | + | 1,476 | 1476 | 11 |
|  |  |  |  |  |  |  |  |
| *Physcomitrella patens* |  |  |  |  |  |  |  |
| [116273](http://www.phytozome.net/genePage.php?search=1&detail=1&crown&method=0&searchText=transcriptid%3A1917138) | *PpZIFL1* | scaffold_15 | 520309 - 525256 | + | 1,329 | 442 | 10 |
| [185929](http://www.phytozome.net/genePage.php?search=1&detail=1&crown&method=0&searchText=transcriptid%3A1888084) | *PpZIFL2* | scaffold_88 | 594417 - 600663 | + | 1,521 | 506 | 12 |
|  |  |  |  |  |  |  |  |

^a^ Previously annotated by Haydon and Cobbett [34]. CDS: predicted coding sequence. TM: transmembrane domains.
