# Supplementary material for: ZINC-INDUCED FACILITATOR-LIKE family in plants: lineage-specific expansion in monocotyledons and conserved genomic and expression features among rice (Oryza sativa) paralogs
Source: BMC Plant Biol. 2011 Jan 25;11:20. doi: 10.1186/1471-2229-11-20 (PMC3041735; doi:10.1186/1471-2229-11-20)
Supplement: Additional File 2 — Previously unannotated ZIFL genes. Gene locus number, given name, chromosome number, genomic localization, strand, predicted coding sequence (CDS) and protein length, exon positions and predicted number of transmembrane domains (TM) are shown for each gene. [file 1471-2229-11-20-S2.DOC]

**Additional File 2.** Previously unannotated *ZIFL* genes.

| Gene **name** | **Chromosome number** | **Locus localizations** | **Strand** | **CDS size (base pairs)** | **Protein size (amino acids)** | **Exon positions^A^** | **TM domains** |
| --- | --- | --- | --- | --- | --- | --- | --- |
|  |  |  |  |  |  |  |  |
| *ZmZIFL1* | 4 | 185583768 - 185587694 | -  (minus) | 1560 | 519 | 185583768 - 185583871; 185584010 - 185584053; 185584209 - 185584297; 185584395 - 185584439; 185584529 - 185584636; 185584719 - 185584804; 185585075 - 185585162; 185585499 - 185585699; 185585808 - 185585883; 185585964 - 185586028; 185586120 - 185586182; 185586266 - 185586319; 185586419 - 185586521; 185586664 - 185586766; 185586897 - 185586957; 185587095 - 185587137; 185587468 - 185587694 | 11 |
| *ZmZIFL8* | 1 | 199261494 - 199264659 | +  (plus) | 1296 | 431 | 199261494 - 199261554; 199261765 - 199261867; 199261963 - 199262065; 199262156 - 199262209; 199262323 - 199262385; 199262473 - 199262522; 199262606 - 199262681; 199262864 - 199263064; 199263227 - 199263314; 199263493 - 199263578; 199263895 - 199264002; 199264057 - 199264126; 199264192 - 199264276; 199264391 - 199264434; 199264556 - 199264659 | 10 |

^A^ For the genes encoded by the minus strand, exon positions are given in the same order they appear in the genomic sequence.

CDS: predicted coding sequence. TM: transmembrane domains.
