# Supplementary figures and images for: ZINC-INDUCED FACILITATOR-LIKE family in plants: lineage-specific expansion in monocotyledons and conserved genomic and expression features among rice (Oryza sativa) paralogs
Source: BMC Plant Biol. 2011 Jan 25;11:20. doi: 10.1186/1471-2229-11-20 (PMC3041735; doi:10.1186/1471-2229-11-20)

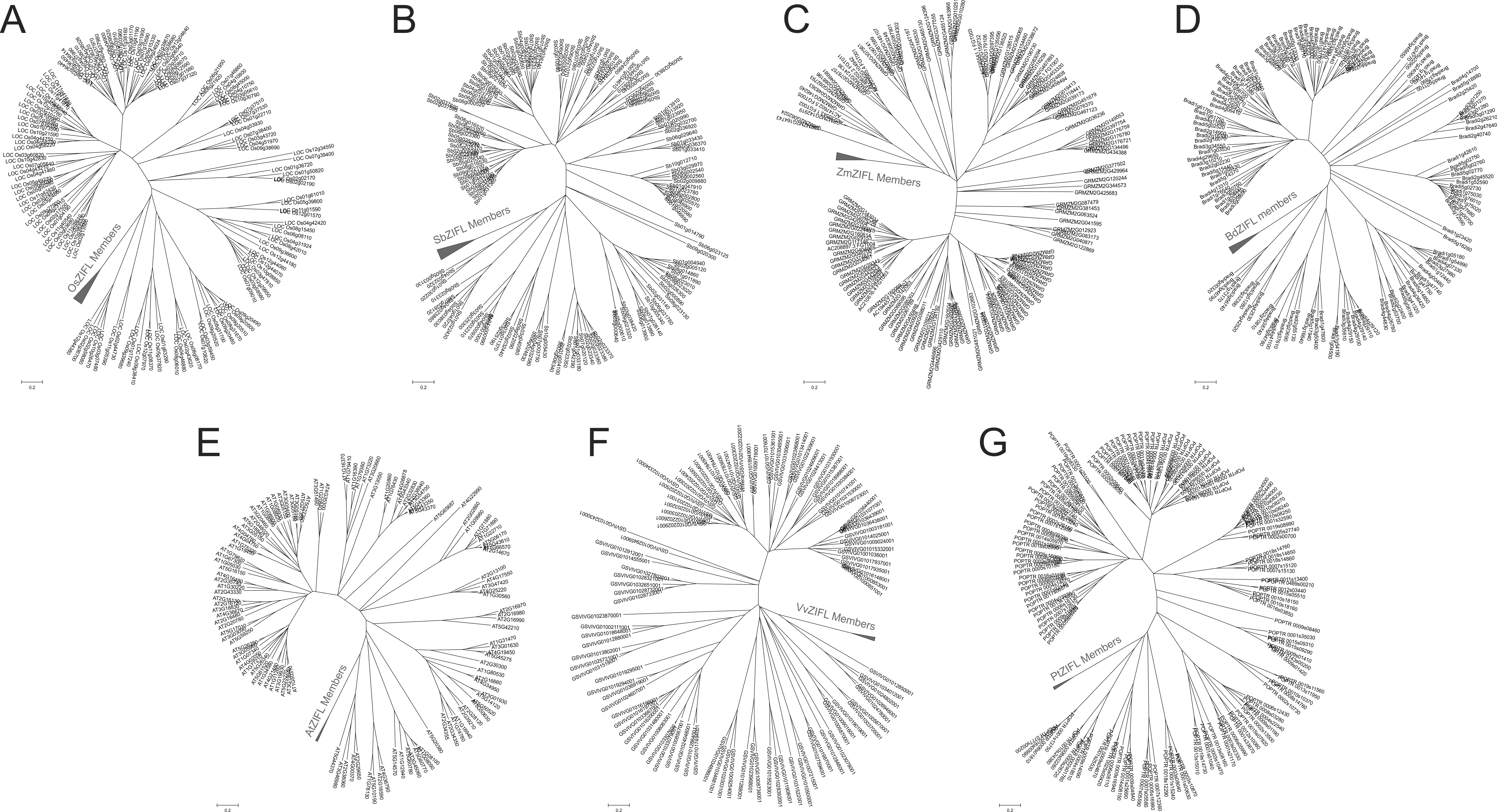

Supplement: Additional File 3 — Phylogenetic trees of ZIFL and MFS_1 proteins. Phylogenetic trees showing the separation of ZIFL proteins from the other MFS_1 sequences in each monocot and dicot species analyzed. (A) Oryza sativa, (B) Sorghum bicolor, (C) Zea mays, (D) Brachypodium distachyon, (E) Arabidopsis thaliana, (F) Vitis vinifera, (G) Populus trichocarpa. [file 1471-2229-11-20-S3.JPEG]

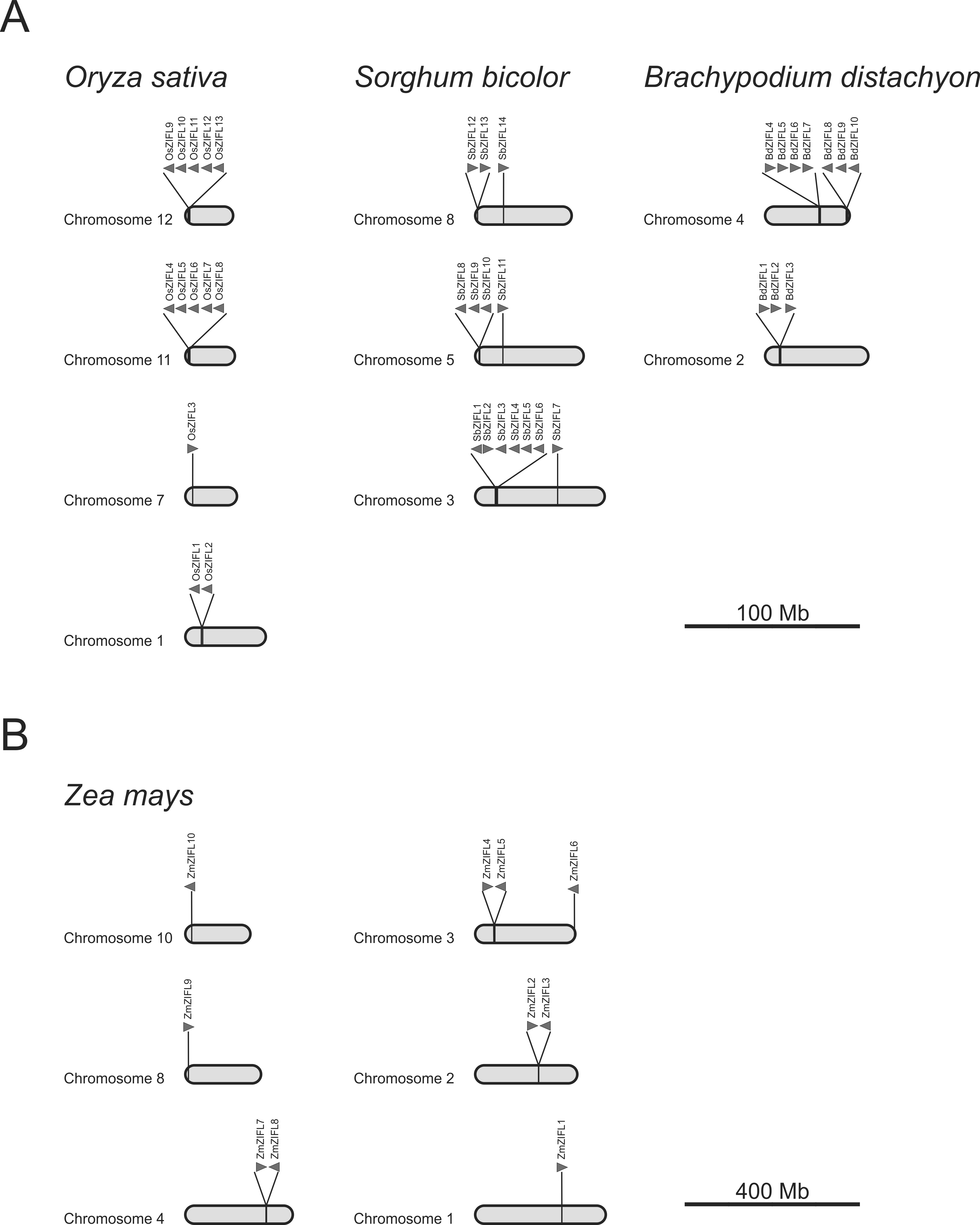

Supplement: Additional File 5 — Chromosomal positions of ZIFL genes. Chromosomal positions of ZIFL genes in (A) Oryza sativa, Sorghum bicolor and Brachypodium distachyon chromosomes, and in (B) Zea mays chromosomes. Only ZIFL-containing chromosomes are shown. Non-ZIFL genes within ZIFL gene clusters were omitted. [file 1471-2229-11-20-S5.JPEG]

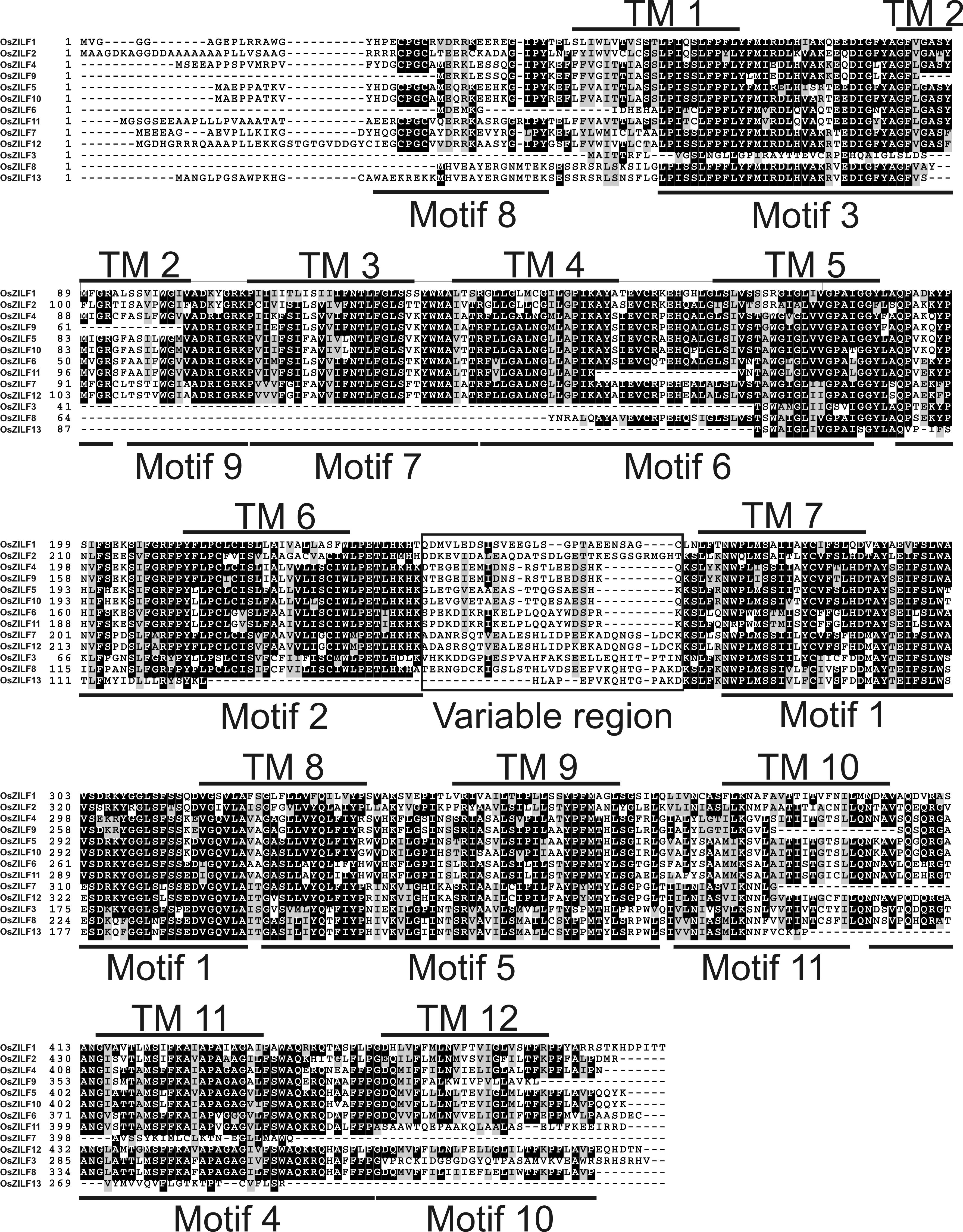

Supplement: Additional File 6 — Alignment of OsZIFL protein sequences. Alignment was constructed using ClustalW. Conserved amino acids are marked in black or grayscale according to similarity level. [file 1471-2229-11-20-S6.JPEG]
