# Supplementary material for: ZINC-INDUCED FACILITATOR-LIKE family in plants: lineage-specific expansion in monocotyledons and conserved genomic and expression features among rice (Oryza sativa) paralogs
Source: BMC Plant Biol. 2011 Jan 25;11:20. doi: 10.1186/1471-2229-11-20 (PMC3041735; doi:10.1186/1471-2229-11-20)
Supplement: Additional File 4 — Conserved residues found in ZIFL protein sequences. Residues of cysteine (Cys) motif, histidine (His) motif, TM8-TM9 loop motif, and residues of MFS and antiporter signatures of each ZIFL protein are shown. [file 1471-2229-11-20-S4.DOC]

**Additional File 4.** Conserved residues found in ZIFL protein sequences.

| **Locus number** | **Gene name** | **Cys Motif**^a^ | **His Motif**^b^ | **TM8-TM9 Loop** | **MFS signature**^d^ | **Antiporter signature**^e^ |
| --- | --- | --- | --- | --- | --- | --- |
|  |  |  |  |  |  |  |
| *Zea mays* |  |  |  |  |  |  |
| [GRMZM2G141081](http://www.phytozome.net/genePage.php?search=1&detail=1&crown&method=0&searchText=transcriptid%3A16930131) | *ZmZIFL2* | CPGC | PETLHKH | KILGPIKTSR | WGMAADRFGRK | SLVGTAWGIGLIIGPALGG |
| [GRMZM2G115658](http://www.phytozome.net/genePage.php?search=1&detail=1&crown&method=0&searchText=transcriptid%3A16924600) | *ZmZIFL3* | CPGC | PETLHRH | KVLGPIKSSR | WGIAADRIGRK | SLVSTAWGIGLIIGPALGG |
| [GRMZM2G029219](http://www.phytozome.net/genePage.php?search=1&detail=1&crown&method=0&searchText=transcriptid%3A16905590) | *ZmZIFL4* | CPGC | PETLHKH | KTVDHITLVR | WGIVADKYGRK | AVVSSSRGIGLIIGPAIGG |
| [GRMZM2G161310](http://www.phytozome.net/genePage.php?search=1&detail=1&crown&method=0&searchText=transcriptid%3A16934500) | *ZmZIFL5* | CPGC | PETLHFH | KYFGPIKTFR | WGMFADKYGRK | SLVTSSRAIALVIGPAIGG |
| [GRMZM2G075594](http://www.phytozome.net/genePage.php?search=1&detail=1&crown&method=0&searchText=transcriptid%3A16915818) | *ZmZIFL6* | CPGC | PETLHFH | RILGTVNSAR | WGVIADRVGRK | SLVNTAWALGLIVGPALGG |
| [GRMZM2G022375](http://www.phytozome.net/genePage.php?search=1&detail=1&crown&method=0&searchText=transcriptid%3A16904120) | *ZmZIFL7* | CPGC | PETLHKH | KILGPVNSTR | WGVVADRVGRK | SVVSTAWGMGVIIGPAIGG |
| [GRMZM2G456923](http://www.phytozome.net/genePage.php?search=1&detail=1&crown&method=0&searchText=transcriptid%3A16948177) | *ZmZIFL9* | CPGC | PETLHFH | KYFGPIKTFR | WGMFADKYGRK | SLVTSSRAIALVIGPALGG |
| [GRMZM2G311401](http://www.phytozome.net/genePage.php?search=1&detail=1&crown&method=0&searchText=transcriptid%3A16921517) | *ZmZIFL10* | - | ^a^PETLHFH^c^ | KYFGPVKIFR | WGIFADKYGRK | SFVTSSRAIALVIGPSIGG |
|  |  |  |  |  |  |  |
| *Oryza sativa* |  |  |  |  |  |  |
| [LOC_Os01g16260](http://www.phytozome.net/genePage.php?search=1&detail=1&crown&method=0&searchText=transcriptid%3A16831123) | *OsZIFL1* | CPGC | PETLHKH | KSVEPITLVR | WGIVADKYGRK | SLVSSSRGIGLIVGPAIGG |
| [LOC_Os01g17214](http://www.phytozome.net/genePage.php?search=1&detail=1&crown&method=0&searchText=transcriptid%3A16831236) | *OsZIFL2* | CPGC | PETLHMH | KYVGPIKPFR | WGIFADKYGRK | SLVTSSRAIALVVGPAIGG |
| [LOC_Os07g08300](http://www.phytozome.net/genePage.php?search=1&detail=1&crown&method=0&searchText=transcriptid%3A16874221) | *OsZIFL3* | - | PETLHDL | KILGPINTSR | - | SLDSTSWAMGLIIGSVIGG |
| [LOC_Os11g04020](http://www.phytozome.net/genePage.php?search=1&detail=1&crown&method=0&searchText=transcriptid%3A16887753) | *OsZIFL4* | CPGC | PETLHKH | KFLGSINSSR | WGVVADRIGRK | SIVSTGWGVGLVVGPAIGG |
| [LOC_Os11g04030](http://www.phytozome.net/genePage.php?search=1&detail=1&crown&method=0&searchText=transcriptid%3A16887754) | *OsZIFL5* | CPGC | PETLHKH | KILGPINSTR | WGMVADRIGRK | SIVSTAWGIGLVVGPAIGG |
| [LOC_Os11g04060](http://www.phytozome.net/genePage.php?search=1&detail=1&crown&method=0&searchText=transcriptid%3A16887760) | *OsZIFL6* | - | PETIHKH | KFLGPIISLR | WGVVADRIGRK | SIVNTAWGLGLVVGPALGG |
| [LOC_Os11g04104](http://www.phytozome.net/genePage.php?search=1&detail=1&crown&method=0&searchText=transcriptid%3A16892118) | *OsZIFL7* | CPGC | PETLHKH | KVIGHIKASR | WGIAADRIGRK | SLVSTAWGIGLIIGPAIGG |
| [LOC_Os11g04150](http://www.phytozome.net/genePage.php?search=1&detail=1&crown&method=0&searchText=transcriptid%3A16887769) | *OsZIFL8* | - | PETLHKH | KVLGIINTSR | - | SLVSTSWAIGLIVGPAIGG |
| [LOC_Os12g03830](http://www.phytozome.net/genePage.php?search=1&detail=1&crown&method=0&searchText=transcriptid%3A16892540) | *OsZIFL9* | - | PETLHKH | KFLGSINSSR | ---VADRIGRK | SIVSTGWGIGLVVGPAIGG |
| [LOC_Os12g03860](http://www.phytozome.net/genePage.php?search=1&detail=1&crown&method=0&searchText=transcriptid%3A16892543) | *OsZIFL10* | CPGC | PETLHKH | KILGPIHSTR | WGMVADRIGRK | SIVSTAWGIGLVVGPATGG |
| [LOC_Os12g03870](http://www.phytozome.net/genePage.php?search=1&detail=1&crown&method=0&searchText=transcriptid%3A16892550) | *OsZIFL11* | CPGC | PETIHKH | KFLGPIISLR | WGVVADRIGRK | --VNTAWGLGLVVGPALGG |
| [LOC_Os12g03899](http://www.phytozome.net/genePage.php?search=1&detail=1&crown&method=0&searchText=transcriptid%3A16892554) | *OsZIFL12* | CPGC | PETLHKH | KVIGHIKASR | WGIAADRIGRK | SLVSTAWGIGLIIGPAIGG |
| [LOC_Os12g03950](http://www.phytozome.net/genePage.php?search=1&detail=1&crown&method=0&searchText=transcriptid%3A16892559) | *OsZIFL13* | - | - | KVLGIINTSR | - | ----TSWAIGLIVGPAISG |
|  |  |  |  |  |  |  |
| *Sorghum bicolor* |  |  |  |  |  |  |
| [Sb03g010620](http://www.phytozome.net/genePage.php?search=1&detail=1&crown&method=0&searchText=transcriptid%3A1961304) | *SbZIFL1* | CPGC | PETLHKH | KAVDHITLVR | WGIVADKYGRK | AVVSSSRGIGLIIGPAIGG |
| [Sb03g011240](http://www.phytozome.net/genePage.php?search=1&detail=1&crown&method=0&searchText=transcriptid%3A1961377) | *SbZIFL2* | CPGC | PETLHFH | KSFGPIRPLR | WGIFADKYGRK | SLVTSSRAIALVIGPAIGG |
| [Sb03g011330](http://www.phytozome.net/genePage.php?search=1&detail=1&crown&method=0&searchText=transcriptid%3A1961389) | *SbZIFL3* | CPGC | PETLHFH | RYFGPIRPLR | WGIFADKYGRK | SLVTSSVAAGLVVGPAIGG |
| [Sb03g011340](http://www.phytozome.net/genePage.php?search=1&detail=1&crown&method=0&searchText=transcriptid%3A1961391) | *SbZIFL4* | CPGC | - | KYFGPIRPLR | WGIFADKYGRK | SLVTSSRAIAFVVGPAIGG |
| [Sb03g011360](http://www.phytozome.net/genePage.php?search=1&detail=1&crown&method=0&searchText=transcriptid%3A1961395) | *SbZIFL5* | CPGC | PETLHFH | KYVGPIKTFR | WGMFADKYGRK | SLVTSSRAIALVIGPAIGG |
| [Sb03g011490](http://www.phytozome.net/genePage.php?search=1&detail=1&crown&method=0&searchText=transcriptid%3A1961413) | *SbZIFL6* | - | PETLHFH | K-FGPIKTFR | WGMFADKYGRK | SFVTSSRAIALVIGPSIGG |
| [Sb03g023750](http://www.phytozome.net/genePage.php?search=1&detail=1&crown&method=0&searchText=transcriptid%3A1962017) | *SbZIFL7* | CPGC | - | KYFGPIRPLR | WGIFADK---- | IKVTSSRAIAFVVGPAIGG |
| [Sb05g002050](http://www.phytozome.net/genePage.php?search=1&detail=1&crown&method=0&searchText=transcriptid%3A1969051) | *SbZIFL8* | CPGC | - | KVLGPINSTR | WGVAADRVGRK | SVVSTAWGMGVIIGPALGG |
| [Sb05g002060](http://www.phytozome.net/genePage.php?search=1&detail=1&crown&method=0&searchText=transcriptid%3A1969052) | *SbZIFL9* | CPGC | PETLHKH | KVLGPIKSSR | WGIAADRIGRK | SLVSTAWGIGLIIGPALGG |
| [Sb05g002070](http://www.phytozome.net/genePage.php?search=1&detail=1&crown&method=0&searchText=transcriptid%3A1969054) | *SbZIFL10* | CPGC | - | - | WGIAADRFGRK | --VGTAWGIGLIIGPALGG |
| [Sb05g008475](http://www.phytozome.net/genePage.php?search=1&detail=1&crown&method=0&searchText=transcriptid%3A1969903) | *SbZIFL11* | CPGC | QETLHTH | KILGPVSTSR | WGMVADRIGRK | SLISTSWAIGLILGPSIGG |
| [Sb08g001400](http://www.phytozome.net/genePage.php?search=1&detail=1&crown&method=0&searchText=transcriptid%3A1977265) | *SbZIFL12* | CPGC | PETLHKH | KVLGPVNASR | WGMIADRIGRK | TLVSTSWAIGLIIGPAIGG |
| [Sb08g001410](http://www.phytozome.net/genePage.php?search=1&detail=1&crown&method=0&searchText=transcriptid%3A1977268) | *SbZIFL13* | CPGC | PETIHKH | - | WGILADRIGRK | --VNTAWALGLIVGPALGG |
| [Sb08g008410](http://www.phytozome.net/genePage.php?search=1&detail=1&crown&method=0&searchText=transcriptid%3A1978062) | *SbZIFL14* | CPGC | PETLHTH | - | WGVVADRIGRK | SIVSTAWGLGLIVGPSIGG |
|  |  |  |  |  |  |  |
| *Brachypodium distachyon* |  |  |  |  |  |  |
| [Bradi2g10020](http://www.phytozome.net/genePage.php?search=1&detail=1&crown&method=0&searchText=transcriptid%3A16482037) | *BdZIFL1* | CPGC | ^a^PETLHKH^c^ | KSIEPIALVR | WGVVADKYGRK | SLISSSRGIGLIVGPAIAG |
| [Bradi2g10030](http://www.phytozome.net/genePage.php?search=1&detail=1&crown&method=0&searchText=transcriptid%3A16482038) | *BdZIFL2* | CPGC | PETLHKH | - | WGVVADKYGRK | SLISSSRGIGLIVGPAIGG |
| [Bradi2g10800](http://www.phytozome.net/genePage.php?search=1&detail=1&crown&method=0&searchText=transcriptid%3A16482129) | *BdZIFL3* | CPGC | PETLHMH | KYAGLIKPFR | WGMFADKYGRK | SLVTSSRAIALVVGPAIGG |
| [Bradi4g26340](http://www.phytozome.net/genePage.php?search=1&detail=1&crown&method=0&searchText=transcriptid%3A16498224) | *BdZIFL4* | CPGC | PETLHNH | GVLGPINTSR | WGMIADRIGRK | SLVSTSWAIGLIVGPTIGG |
| [Bradi4g26350](http://www.phytozome.net/genePage.php?search=1&detail=1&crown&method=0&searchText=transcriptid%3A16498225) | *BdZIFL5* | CPGC | PETLHNH | - | WGVIADRIGRK | SLVSTSWAIGLIIGPTIGG |
| [Bradi4g26370](http://www.phytozome.net/genePage.php?search=1&detail=1&crown&method=0&searchText=transcriptid%3A16498228) | *BdZIFL6* | CPGC | PETLHKH | KVLGHVRSSQ | WGIAADRIGRK | SLVSTAWGIGLIIGPALGG |
| [Bradi4g26380](http://www.phytozome.net/genePage.php?search=1&detail=1&crown&method=0&searchText=transcriptid%3A16498232) | *BdZIFL7* | CPGC | PETLHMH | RYLGSIISSR | WGVVADRIGRK | SVVSTGWGIGLVAGPAIGG |
| [Bradi4g43580](http://www.phytozome.net/genePage.php?search=1&detail=1&crown&method=0&searchText=transcriptid%3A16500363) | *BdZIFL8* | CPGC | PESLHKH | KIFGPINLTR | WGMVADRIGRK | STVNTAWGIGLIIGPAIGG |
| [Bradi4g43590](http://www.phytozome.net/genePage.php?search=1&detail=1&crown&method=0&searchText=transcriptid%3A16500365) | *BdZIFL9* | CPGC | PETIHKH | KFLGPVISAR | WGIVADRIGRK | SVVNTAWGFGLVIGPALGG |
| [Bradi4g43620](http://www.phytozome.net/genePage.php?search=1&detail=1&crown&method=0&searchText=transcriptid%3A16500370) | *BdZIFL10* | - | PETIHKH | KFLGPLISSR | WGIAADRLGRK | SVVNTAWGVGLVIGPGLGG |
|  |  |  |  |  |  |  |
| *Arabidopsis thaliana* |  |  |  |  |  |  |
| [AT5G13740](http://www.phytozome.net/genePage.php?search=1&detail=1&crown&method=0&searchText=transcriptid%3A17373643) | - | CPGC | PETLHNH | KLLGPVLVTR | WGIVADRYGRK | SAVSTAWGIGLIIGPALGG |
| [AT5G13750](http://www.phytozome.net/genePage.php?search=1&detail=1&crown&method=0&searchText=transcriptid%3A17373644) | - | CSGC | PETLHNH | RLLGPIIVTR | WGLVADRYGRK | SAVSTAWGIGLIIGPAIGG |
| [AT3G43790](http://www.phytozome.net/genePage.php?search=1&detail=1&crown&method=0&searchText=transcriptid%3A17364550) | - | CPGC | PETLHTR | KSVGLLAVIR | WGKLADRYGRK | SVVSTSRGIGLILGPAIGG |
|  |  |  |  |  |  |  |
| *Populus trichocarpa* |  |  |  |  |  |  |
| [POPTR_0006s02710](http://www.phytozome.net/genePage.php?search=1&detail=1&crown&method=0&searchText=transcriptid%3A17342279) | *PtZIFL1* | CPGC | PETLHSH | RNFGPVMVSR | WGMIADRYGRK | SIISTSWGIGLVIGPALGG |
| [POPTR_0008s01110](http://www.phytozome.net/genePage.php?search=1&detail=1&crown&method=0&searchText=transcriptid%3A17316236) | *PtZIFL2* | CPGC | PETLHVH | RILGPIPVAQ | WGIVADRYGRK | STVSTAWGLGLIIGPALGG |
| [POPTR_0009s02680](http://www.phytozome.net/genePage.php?search=1&detail=1&crown&method=0&searchText=transcriptid%3A17332056) | *PtZIFL3* | CPGC | PETLHMH | KVLGPITVTR | WGWLADRYGRK | SVVSTSRGIGMIIGPAIGG |
| [POPTR_0016s02500](http://www.phytozome.net/genePage.php?search=1&detail=1&crown&method=0&searchText=transcriptid%3A17325150) | *PtZIFL4* | CPGC | PETIHNH | RNFGPVMVSR | WGMIADRYGRK | SIISTSWGIGLVIGPALGG |
|  |  |  |  |  |  |  |
| *Vitis vinifera* |  |  |  |  |  |  |
| [GSVIVT00023334001](http://www.phytozome.net/genePage.php?search=1&detail=1&crown&method=0&searchText=transcriptid%3A1724285) | *VvZIFL1* | CPGC | QETLHIH | RILGPVMVSR | WGMVADRYGRK | SIISTAWGIGLVIGPALGG |
| [GSVIVT00033966001](http://www.phytozome.net/genePage.php?search=1&detail=1&crown&method=0&searchText=transcriptid%3A1734917) | *VvZIFL2* | CPGC | QETLHTH | KLLGPIIICR | WGMVADRYGRK | STINTAWGVGLIIGPALGG |
| [GSVIVT00033971001](http://www.phytozome.net/genePage.php?search=1&detail=1&crown&method=0&searchText=transcriptid%3A1734922) | *VvZIFL3* | CPGC | QETLHTH | KLLGPIIICR | WGMVADRYGRK | STINTAWGVGLIIGPALGG |
| [GSVIVT00033972001](http://www.phytozome.net/genePage.php?search=1&detail=1&crown&method=0&searchText=transcriptid%3A1734923) | *VvZIFL4* | CSGC | PETLHTH | RFLGPVMICR | WGMVADRYGRK | STVSTARGIGLIIGPALGG |
| [GSVIVT00033975001](http://www.phytozome.net/genePage.php?search=1&detail=1&crown&method=0&searchText=transcriptid%3A1734926) | *VvZIFL5* | CSGC | PETLHTH | RFLGPVMICR | WGMVADRYGRK | STVSTAWGIGLIIGPALGG |
|  |  |  |  |  |  |  |
| *Selaginella moellendorffii* |  |  |  |  |  |  |
| [164091](http://www.phytozome.net/genePage.php?search=1&detail=1&crown&method=0&searchText=transcriptid%3A15407448) | *SmZIFL1* | CPGC | PETLHRK | RMVGPIRACR | WGSIADRYGRK | SVVGTAWGLGLIIGPAVGG |
| [408219](http://www.phytozome.net/genePage.php?search=1&detail=1&crown&method=0&searchText=transcriptid%3A15420283) | *SmZIFL2* | CPGC | PETLHRK | RLLGPIRACR | WGSIADRYGRK | SVVGTAWGFGLIVGPALGG |
| [147893](http://www.phytozome.net/genePage.php?search=1&detail=1&crown&method=0&searchText=transcriptid%3A15407953) | *SmZIFL3* | CPGC | PETLHKD | KWLGPIRLTR | WGVIADRYGRR | SMVSTMWGFGLIIGPAMGG |
| [96275](http://www.phytozome.net/genePage.php?search=1&detail=1&crown&method=0&searchText=transcriptid%3A15406286) | *SmZIFL4* | CPGC | PETLHKH | NMLGPIFMTR | WGYLADRIGRR | SVVGTTWGLGLIVGPALGG |
| [412524](http://www.phytozome.net/genePage.php?search=1&detail=1&crown&method=0&searchText=transcriptid%3A15412271) | *SmZIFL5* | CPGC | PETLHIH | RRLGPAMVTR | WGVAADRYGRK | - |
| [157948](http://www.phytozome.net/genePage.php?search=1&detail=1&crown&method=0&searchText=transcriptid%3A15415971) | *SmZIFL6* | CPGC | PETLHKH | RLIGAIRSCR | WGRIADKYGRK | STVGTAWGLGLIIGPSLGG |
| [184037](http://www.phytozome.net/genePage.php?search=1&detail=1&crown&method=0&searchText=transcriptid%3A15421729) | *SmZIFL7* | CPGC | PETLHKH | RLIGAIRSYR | WGRIADKYGRK | STVGTTWGLGLIVGPSLGG |
|  |  |  |  |  |  |  |
| *Physcomitrella patens* |  |  |  |  |  |  |
| [116273](http://www.phytozome.net/genePage.php?search=1&detail=1&crown&method=0&searchText=transcriptid%3A1917138) | *PpZIFL1* | - | - | NWMGAVLVSR | WGMVADKYGRV | SIVGTVWGLGLIIGPAMGG |
| [185929](http://www.phytozome.net/genePage.php?search=1&detail=1&crown&method=0&searchText=transcriptid%3A1888084) | *PpZIFL2* | CPGC | - | HWMGAVLVSR | WGMASDRYGRK | SIVGTVWGLGLIIGPAMGG |
|  |  |  |  |  |  |  |

^a^ Only the most frequent C-P-G-C residues are highlighted.

^b^ Only the most frequent P-E-T-L-H-x-H residues are highlighted.

^c^ There is a sequence gap between the first and second positions.

^d^ Only the canonical MFS G-x(3)-D-[RK]-x-G-R-[RK] residues are highlighted.

^e^ Only the Antiporter S-x(8)-G-x(3)-G-P-x(2)-G-G residues are highlighted.
