# Supplementary material for: ZINC-INDUCED FACILITATOR-LIKE family in plants: lineage-specific expansion in monocotyledons and conserved genomic and expression features among rice (Oryza sativa) paralogs
Source: BMC Plant Biol. 2011 Jan 25;11:20. doi: 10.1186/1471-2229-11-20 (PMC3041735; doi:10.1186/1471-2229-11-20)
Supplement: Additional File 7 — Probes used in Genevestigator analyses and evaluation of specificity of rice ZIFL Affymetrix® microarray probes. For each ZIFL gene locus number, all corresponding probes are listed. Each probe is classified as unique or not unique, and, in the second case, the number of the other locus matching to the probe is provided. [file 1471-2229-11-20-S7.DOC]

**Additional File 7.** Probes used in Genevestigator analyses and evaluation of specificity of rice ZIFL Affymetrix® microarray probes.

| **Gene** | **Probe** | **Specificity** | **Other match** |
| --- | --- | --- | --- |
| LOC_Os01g16260 | Os.10292.1.S1_at | Not unique | LOC_Os01g16250 |
|  | Os.39764.1.S1_at | Not unique | LOC_Os01g16250 |
|  | Os.39764.1.S1_x_at | Not unique | LOC_Os01g16250 |
|  |  |  |  |
| LOC_Os01g17214 | Os.17416.1.S1_at^*^ | Unique (*OsZIFL2*) | - |
|  | OsAffx.22176.1.S1_x_at^*^ | Unique | - |
|  |  |  |  |
| LOC_Os07g08300 | Os.16958.1.S1_at | Unique (*OsZIFL3*) | - |
|  |  |  |  |
| LOC_Os11g04020 | Os.18707.1.S1_at | Not unique | LOC_Os12g03830 |
|  |  |  |  |
| LOC_Os11g04030 | Os.6142.1.S1_s_at | Unique (*OsZIFL5*) | - |
|  |  |  |  |
| LOC_Os11g04104 | Os.18177.1.S1_at | Unique (*OsZIFL7*) | - |
|  | Os.9752.2.S1_x_at | Not unique | LOC_Os12g03899 |
|  | Os.9752.3.S1_x_at | Not unique | LOC_Os12g03899 |
|  |  |  |  |
| LOC_Os11g04150 | OsAffx.19478.1.S1_at | Unique (*OsZIFL8*) | - |
|  | OsAffx.19478.2.S1_s_at | Not unique | LOC_Os12g03950 |
|  |  |  |  |
| LOC_Os12g03830 | No probe |  |  |
|  |  |  |  |
| LOC_Os12g03860 | Os.21635.1.S1_at | Unique (*OsZIFL10*) | - |
|  |  |  |  |
| LOC_Os12g03899 | Os.9752.1.S1_a_at | Unique (*OsZIFL12*) | - |
|  | Os.9752.2.S1_x_at | Not unique | LOC_Os11g04104 |
|  | Os.9752.3.S1_x_at | Not unique | LOC_Os11g04104 |
|  |  |  |  |
| LOC_Os12g03950 | OsAffx.19478.2.S1_at | Unique (*OsZIFL13*) | - |
|  | OsAffx.19478.2.S1_s_at | Not unique | LOC_Os11g04150 |
|  |  |  |  |

^*^ Distinct probes for the same gene, with the same expression pattern.

Probes used in Figures 12 and 13 are indicated by the respective ZIFL gene names between brackets (third column).
