# Supplementary material for: ZINC-INDUCED FACILITATOR-LIKE family in plants: lineage-specific expansion in monocotyledons and conserved genomic and expression features among rice (Oryza sativa) paralogs
Source: BMC Plant Biol. 2011 Jan 25;11:20. doi: 10.1186/1471-2229-11-20 (PMC3041735; doi:10.1186/1471-2229-11-20)
Supplement: Additional File 9 — Gene-specific primers used for quantitative RT-PCR. Sequences of PCR primers used in quantitative RT-PCR analyses of rice ZIFL gene expression. [file 1471-2229-11-20-S9.DOC]

Additional File 9: Gene-specific primers used for quantitative RT-PCR.

| **Gene name ^a^** | **Forward Primer 5’ → 3’** | **Reverse Primer 5’ → 3’** |
| --- | --- | --- |
| *OsZIFL1* | GAAGAAGCACGAAGCACGAT | AAAAATGGGGGTCTACATCAGA |
| ***OsZIFL2*** | GCCTGGATTCTAACCTGCAA | TGGTTCGGAGTTCAGACAGA |
| ***OsZIFL3*** | GATCTGGAGTTGTGCCCAATA | TCATATGGCGAAGGAAAGAA |
| ***OsZIFL4*** | TGTGATTGAATTAATTGGACTTGC | GGGGTGCTATTCCAGCTTCT |
| ***OsZIFL5*** | TTGTAGCAGAGTGTTAGAAACATGC | GCAGAACAAAAAGAAGAACAACC |
| *OsZIFL6* | TGCCTTCTTCTTTCCAGGTG | GCCGCAGGTAACACCATAAA |
| ***OsZIFL7*** | CTGTGAAGCGAAGGCATATAA | TATACAAGCATAAGGAGGTACCAA |
| *OsZIFL8* | ATCAAAGAGCAACCGCAAAT | CGAAAACAGAATGCCTGCTC |
| ***OsZIFL9*** | TGCCCTAAAATGGATTGTCC | CAATGTTGATCTGACCCCAAA |
| ***OsZIFL10*** | TTACAGTGAGTAATAAGTATGGCTTGG | AAAGCAGAACAAAATCCAACAGA |
| *OsZIFL11* | CCCTCTTCTTTCCAGCTTCA | TTAGTCCCGCCGTATCTCCT |
| ***OsZIFL12*** | CGCTGTGTGAAGCAAAGGTA | GCTTTGATTCTTTGGGACAGA |
| *OsZIFL13* | CTCAAGACCTTGGTTGTCCA | CCATGTACACCGGTAGTTTGC |

^a^ Gene names in bold are of *OsZIFL* genes which show expression in at least one qPCR experiment.
